# A Comprehensive Systematic Review and Meta‐Analysis on the Prevalence of Aflatoxin M1 in Dairy Products in Selected Middle East Countries

**DOI:** 10.1002/vms3.70204

**Published:** 2025-01-22

**Authors:** Bahareh Arghavan, Kosar Kordkatuli, Helia Mardani, Ali Jafari

**Affiliations:** ^1^ Department of Basic Medical Sciences School of Medicine Abadan University of Medical Sciences Abadan Iran; ^2^ Student Research Committee Department of Surgical Technology School of Paramedical Sciences Golestan University of Medical Sciences Gorgan Iran; ^3^ Student Research Committee Department of Nutrition School of Nutritional Sciences and Dietetics Tehran University of Medical Sciences (TUMS) Tehran Iran; ^4^ Student Research Committee Department of Community Nutrition Faculty of Nutrition Sciences and Food Technology National Nutrition and Food Technology Research Institute Shahid Beheshti University of Medical Sciences Tehran Iran; ^5^ Systematic Review and Meta‐analysis Expert Group (SRMEG) Universal Scientific Education and Research Network (USERN) Tehran Iran

**Keywords:** aflatoxin M1, cheese, meta‐analysis, milk, mycotoxins, prevalence

## Abstract

**Background:**

Human consumption of dairy products contaminated with aflatoxin (AF) M1 can lead to severe health issues. This AF's significance and impact on health necessitate a thorough investigation of its prevalence in dairy products.

**Objectives:**

This study aims to determine the prevalence of AFM1 in dairy products through a systematic review and meta‐analysis, focusing on data from Middle Eastern countries.

**Methods:**

We identified relevant studies through electronic database searches (PubMed, Scopus and Web of Science) up to August 2023. We employed a random‐effects model to derive an overall estimate and used 95% confidence intervals to determine pooled prevalence rates.

**Results:**

The meta‐analysis included 193 studies encompassing 297,530 samples of dairy products. The results showed that AFM1 contaminated 87% of dairy products. The following is a ranking of countries based on the prevalence of AFM1 in their dairy products: Iran > Jordan > Turkey > Kuwait > Lebanon > Syria > Egypt > Cyprus > the United Arab Emirates. The current meta‐analysis indicated that Middle Eastern countries exhibited a high prevalence of AFM1 in dairy products. The prevalence rates for AFM1 in various dairy products were as follows: milk (87%), yogurt (68.9%), cheese (63.6%), kashk (62.9%), doogh (55.6%) and ice cream (54%). Pasteurized milk (99.5%), ultra‐high temperature (91.3%), raw milk (73%) and traditional milk (51%), among other milk types, had the highest contamination rates.

**Conclusion:**

The study reveals a high prevalence of AFM1 in dairy products, particularly in Middle Eastern countries. Given the critical importance of milk and dairy products in the diet, special measures are needed to safeguard their quality and protect consumers from AF contamination.

## Graphical abstract

• A high prevalence of aflatoxin M1 was found in dairy products in the Middle Eastern countries: (Iran > Jordan > Turkey > Kuwait > Lebanon > Syria > Egypt > Cyprus > UAE).

• The prevalence of aflatoxin M1 in dairy products, such as milk, yogurt, cheese and ice cream, was 87%, 68.9%, 63.6% and 54%, respectively.

• The prevalence of AFM1 in dairy products from various animal species was classified to goat > cow > sheep > camel.

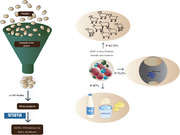

## 1 Introduction

Aflatoxins (AFs) constitute a subgroup of mycotoxins produced by various species of *Aspergillus* fungi (Caceres et al. 2020). These fungi are common and have a wide‐reaching impact, affecting food such as cereals, peanuts, almonds and pistachios. Around 25% of the world's food supply is contaminated with AFs and other mycotoxins at different stages of production and distribution. The frequent presence of AFs in food globally has raised substantial alarm because of their severe toxicity, mutagenic effects, ability to cause birth defects and potential to trigger cancer (Engin and Engin 2019; Fetaih et al. 2014; Groopman et al. 1988; Ostry et al. 2017). Prolonged or chronic exposure to AFs can result in the accumulation of toxins in the body, disrupting the function of many organs, particularly the liver and kidneys (Klingelhöfer et al. 2018). The International Agency for Research on Cancer (IARC) has recently classified AFs as human carcinogens (Ostry et al. 2017). AFB1 is the most potent carcinogenic substance among the various forms of AFs, including B1, B2, G1 and G2 (Frayssinet and Lafarge‐Frayssinet 1990). AFB1 has been categorized as a carcinogen by the World Health Organization due to its involvement in the development of hepatocellular carcinoma (Magnussen and Parsi 2013). AFM1 is a monohydroxylated metabolite formed by the biotransformation of AFB1 in the liver (Frazzoli et al. 2017). AFB1 ingested by lactating animals is subsequently excreted in the milk, where it attaches to casein and remains bound to it during the processing of dairy products, particularly in cheeses (Firmin et al. 2011). Furthermore, the individual's health may be at risk due to increased mycotoxin levels in the body. AFM1 has been classified as one of the most severe chemical compounds in milk products, such as cheese, yogurt, pasteurized and sterilized milk and other dairy products, owing to its high persistence in thermal processing, for example, pasteurized and ultra‐high temperature (UHT) treatment (Danezis et al. 2016; Tsakiris et al. 2013). Due to the potential health hazards associated with AFM1 in milk, more than 60 countries have established maximum permissible levels (Sadia et al. 2012). European Union (EU) regulations specify that the maximum residue level (MRL) for AFM1 in raw and heat‐treated milk is 50 ng/L. However, the United States guidelines have adjusted this level to 500 ng/kg (Campagnollo et al. 2016; The Commission of the European Communities 2004). In recent years, the presence of AFM1 in milk and dairy products worldwide has become a significant concern given the widespread consumption of these foods and the potential risks of AFM1 to human health, particularly in age groups more susceptible to immune‐related issues (Simon, Hollander, and McMichael 2015; Tsakiris et al. 2013). Prolonged exposure to AFM1 can result in immunosuppression, hepatocarcinoma and impaired growth in children. Exposure to AFM1 poses a significant health risk due to its likely interaction with DNA (Zhang et al. 2019).

In recent years, researchers have become significantly more interested in these matters, resulting in the publication of different data on AF contamination of dairy products. Thus, the present study aims to conduct a systematic review and meta‐analysis regarding the prevalence of AFM1 in dairy products, including milk, cheese, yogurt, doogh, kashk and ice cream. Moreover, it assesses the prevalence of AFM1 based on subgroups such as Middle Eastern countries, seasons, animals and different detection methods.

## 2 Methods

We strictly adhered to the guidelines established by the Preferred Reporting Items for Systematic Reviews and Meta‐analyses (PRISMA) to ensure the systematic review and meta‐analysis conducted in this study (Moher et al. 2015). In preparation for this systematic review and meta‐analysis, we registered our study protocol with a recognized PROSPERO registry with the registration number CRD42023461934. This measure was taken to ensure the adherence of our research to predefined methods and objectives and to enhance transparency throughout the research process. Because our investigation entails a methodical examination and meta‐analysis of previously published research, no ethical acquiescence or informed consent was necessary. The regional ethical committee also authorized the study protocol at IR.ABADANUMS.REC.1403.131.

### 2.1 Systematic Search

A comprehensive search was conducted across MEDLINE/PubMed, Web of Science and Scopus databases from inception to August 2023. Additionally, we explored grey literature sources by manually examining bibliographies and internet searches on Google and Google Scholar. The search strategy was meticulously designed to encompass all relevant literature, employing variations of critical terms such as ‘AFM1’, ‘dairy products’ and ‘Middle East’. Table S1 provides a detailed outline of the systematic search strategy employed. Two investigators (XXXXX) independently conducted article searches to minimize bias. In the cases of disagreement, a referee supervisor (XXXXX) reviewed the article in question. All articles were imported into EndNote reference management software X8 (Thompson and Reuters, Philadelphia, USA), and duplicates were meticulously removed.

### 2.2 Inclusion/Exclusion Criteria and Outcomes

We established rigorous study inclusion and exclusion standards to maintain the highest scientific rigor. Studies were considered for inclusion in our investigation only if they met the following criteria: (i) conducted within the geographic boundaries of the Middle East; (ii) reported levels of AFM1 contamination in a wide range of dairy products, including but not limited to milk, cheese, yogurt and others; (iii) utilized validated and accurate analytical methods to quantify AFM1; (iv) provided comprehensive information on study design, sample size and sampling procedures. Conversely, exclusion criteria were applied to exclude duplicate studies, those with unclear methodology, those lacking statistical indices, those that were inaccessible in their entirety, those that failed to meet the inclusion criteria and those not written in English (Table S2).

The primary objective of our meta‐analysis was to determine the prevalence of AFM1 contamination in dairy products from the Middle East. Ancillary objectives included the examination of variations in contamination levels across different categories of dairy products and among various geographic locations within the Middle Eastern region.

### 2.3 Screening and Data Extraction

Two reviewers (XXXXX) independently screened titles, abstracts and full texts in duplicate. Studies that met our inclusion criteria were selected for full‐text examination. Our data extraction process was meticulously executed, utilizing the structured information in the Excel file. To enhance the reliability and accuracy of our findings, two independent reviewers (XXXXX) rigorously conducted the data extraction process. We systematically collected key data points, including the author's name, publication year, city, country, study type, sampling period, seasonal variations, types of dairy products investigated, milk source, cheese type (if applicable), specific cheese names (if mentioned), animal species providing the milk, total number of samples analysed, the number of samples where AFM1 was detected, the calculated prevalence expressed as a percentage, the analytical method employed for AFM1 detection, the range of AFM1 concentrations (in ng/kg), mean AFM1 concentrations (in ng/kg), standard deviation (SD), standard error (SE) when available, the overall range of concentrations, the limit of detection (LOD—in ng/kg) and the limit of quantification (LOQ—in ng/kg). This comprehensive data extraction process ensures a holistic understanding of AFM1 prevalence in Middle Eastern dairy products, facilitating robust meta‐analysis and interpretation. Furthermore, the results of this extraction underwent thorough review and revision under the supervision of the study's design supervisor (XXXXX). We utilized the Joanna Briggs Institute (JBI) critical appraisal checklist to evaluate the quality and potential biases in the included studies. Specifically, this checklist was applied to studies reporting prevalence data (Munn et al. 2015). The evaluation checklist presented here comprises nine items that evaluate various aspects of a research study, including the appropriateness of the sampling frame, the use of proper sampling techniques, the adequacy of the sample size, the description of the study subject and setting, the sufficiency of the data analysis, the use of valid methods for the identified conditions, the validity of the measurement for all participants, the use of appropriate statistical analysis and the response rate. Each item is graded as ‘yes’, ‘no’, ‘unclear’ or ‘not applicable’, with a score of 1 allotted for ‘yes’ and 0 for ‘no’ and ‘unclear’ responses. The mean score is then calculated for each article, and studies with scores below and above the mean are classified as poor and good quality, respectively.

### 2.4 Data Synthesis and Analysis

In our pursuit of a rigorous methodology, we employed Stata statistical software (v.16, Stata Corp., College Station, TX, USA) to conduct all statistical analyses meticulously. Our meta‐analysis calculated the prevalence of AFM1 contamination in Middle Eastern dairy products for each eligible study. This entailed determining the number of positive samples with detectable AFM1 divided by the total number of samples tested, ensuring an accurate representation of AFM1 occurrence. To obtain a more conservative estimate of AFM1 prevalence, we applied the DerSimonian–Laird random‐effects meta‐analysis model (REM) (DerSimonian and Laird 1986), which accounts for potential variations between studies and provides a robust and balanced overall estimate of AFM1 contamination. To evaluate potential heterogeneity among the included studies, we conducted a comprehensive investigation using the *I* ^2^ statistic and Cochran's *Q* test (Higgins et al. 2003). An *I* ^2^ score exceeding 75% would indicate significant heterogeneity, leading us to conduct more in‐depth subgroup analyses. Subgroup analyses were meticulously carried out to explore possible sources of heterogeneity, such as study design, sample size, sampling methods and geographical regions, aligning with our commitment to methodological excellence. To ensure comprehensive coverage of relevant studies, we diligently examined potential publication bias through Egger's test (Egger et al. 1997). Furthermore, we conducted sensitivity analyses to assess the potential impact of individual studies on the overall estimate, enhancing the robustness and reliability of our findings. This comprehensive approach to data synthesis and analysis seamlessly aligns with our study's overarching goals, ensuring the validity and accuracy of our conclusions.

## 3 Results

### 3.1 Study Characteristics

**FIGURE 1:**
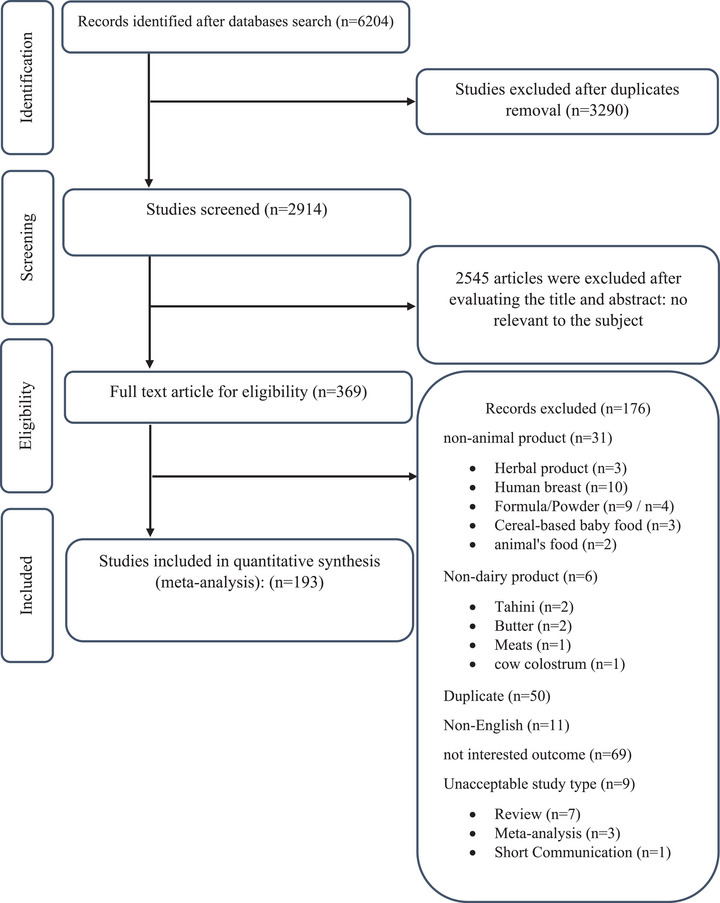
Flowchart of study selection for inclusion trials in the systematic review.

This study systematically reviewed all studies on the prevalence of AFM1 in dairy products in the Middle East, adhering to the PRISMA regulations. Figure 1 presents the selection process of the included studies. Out of the total 6204 articles, the initial literature screening excluded 3290. We selected 2914 articles based on their titles and abstracts as potentially suitable published sources for data. We excluded 2545 articles based on their title and abstract, deeming them irrelevant to the subject. After evaluating the eligibility of 369 articles, we eliminated 176 articles for various reasons: either they reported non‐animal products such as herbal products (*n* = 3), human breast products (*n* = 10), formula/powder (*n* = 9/*n* = 4), cereal‐based baby food (*n* = 3), animal food (*n* = 2) or non‐dairy products like tahini (*n* = 2), butter (*n* = 2), meats (*n* = 1), cow colostrum (*n* = 1) or they contained no original data, such as reviews, meta‐analyses and brief communications. The full‐text review excluded the studies listed in Table S2, along with the reasons for their exclusion. We finally included 193 articles containing 7484 data published from inception to August 2023 in our analysis.

The characteristics of the included studies are presented in Table 1. All studies had a cross‐sectional design and were published between 1990 and 2023. Our investigation across three international databases, including Scopus, PubMed and Web of Science, revealed that only 9 out of 18 Middle Eastern countries had published articles on AFM1 contamination in dairy products. These countries were Iran (94 studies), Turkey (58 studies), Egypt (19 studies), Lebanon (5 studies), Cyprus (3 studies), Jordan (3 studies), Kuwait (2 studies), Syria (2 studies) and United Arab Emirates (2 studies).

**TABLE 1:** Main characteristics of all eligible studies reporting the prevalence of Aflatoxin M1 in dairy products.

Reference	Country	Season	Dairy products	Animal species	Number of samples	Number of positive samples	Range (ng/kg)	Quality assessment Score
Saad, Abdelgadir, and Moss (1989)	United Arab Emirates	NR	Milk	Camel	20	6	250–800	6/9
Haydar, Benelli, and Brera (1990)	Syria	Winter	Cheese	NR	11	1	0–19,000	7/9
Ioannou‐Kakouri et al. (1999)	Cyprus	NR	Milk	NR	106	12	10–40	9/9
Bakirci (2001)	Turkey	Spring	Milk, cream and cheese	NR	183	183	NR	9/9
Ivastava Sr et al. (2001)	Kuwait	Summer	Milk, yoghurt	Cow, sheep, goat and camel	35	14	NR	7/9
Hismiogullari, Basalan, and Hismiogullari (2003)	Turkey	NR	Cheese	NR	46	46	NR	6/9
Gürses, ERDOĞAN, and Çetin (2004)	Turkey	NR	Cheese	NR	63	28	7–202	7/9
Sarımehmetoglu, Kuplulu, and Celik (2004)	Turkey	All	Cheese	NR	400	327	NR	8/9
Kamkar (2005)	Iran	All	Milk	NR	111	85	15–280	9/9
Çelik et al. (2005)	Turkey	NR	Milk	NR	85	75	NR	6/9
Kamber (2005)	Turkey	winter	Cheese	NR	60	10	51.10–115.0	7/9
Aycicek, Aksoy, and Saygi (2005)	Turkey	All	Cheese	NR	196	177	NR	8/9
Yaroglu, Oruc, and Tayar (2005)	Turkey	All	Cheese	NR	600	30	100–800	8/9
Tekinşen and Tekinşen (2005)	Turkey	All	Cheese	NR	110	83	100–72,600	7/9
Alborzi, Rashidi, and Astaneh (2006)	Iran	Spring and summer	Milk	NR	624	624	NR	8/9
Kamkar (2006)	Iran	All	Cheese	NR	80	66	150–2410	7/9
Akkaya et al. (2006)	Turkey	Spring, Summer	Yogurt	NR	177	104	NR	8/9
Gürbay, Engin, et al. (2006)	Turkey	Winter	Yogurt, cheese	NR	79	43	51–365.64	7/9
Bașkaya et al. (2006)	Turkey	All	Cheese	NR	363	340	NR	7/9
Unusan (2006)	Turkey	Winter	Milk	NR	129	129	0–543.64	7/9
Gürbay, Aydın, et al. (2006)	Turkey	NR	Milk	NR	27	16	NR	6/9
Colak et al. (2006)	Turkey	NR	Cheese	NR	24	17	NR	5/9
Tajik, Rohani, and Moradi (2007)	Iran	NR	Milk	NR	144	144	4.3–91.8	8/9
Karimi et al. (2007)	Iran	Spring	Milk	NR	110	110	8–89	8/9
Ghiasian et al. (2007)	Iran	All	Milk	NR	186	119	15–> 410	8/9
Oveisi et al. (2007)	Iran	Spring, summer	Milk	NR	128	128	31–113	7/9
Tajkarimi et al. (2008)	Iran	Winter, summer	Milk	NR	319	172	NR	8/9
Oezdemir (2007)	Turkey	Spring	Milk	Goat	110	93	5.16–116.78	9/9
Ayar, SERT, and Con (2007)	Turkey	Summer, autumn	Milk	NR	48	48	3–200	5/9
Kamkar (2008)	Iran	Summer, autumn	milk	NR	52	52	19.40–93.60	8/9
Tekinşen and Uçar (2008)	Turkey	Spring	Cheese	NR	100	99	0–4100	8/9
Yapar et al. (2008)	Turkey	NR	Cheese	NR	105	75	NR	7/9
Tekinşen and Eken (2008)	Turkey	Autumn, winter	Milk, cheese	NR	232	176	10–690	9/9
Akkaya et al. (2009)	Turkey	Spring, summer	Cheese	NR	242	211	NR	7/9
Motawee, Bauer, and McMahon (2009)	Egypt	Summer	Milk	Buffalo, cow, goat, camel	175	49	NR	8/9
Fallah et al. (2009)	Iran	NR	Cheese	NR	210	161	52.1–785.4	8/9
Ghazani (2009)	Iran	Summer, autumn	Milk	NR	50	50	0–259	8/9
Movassagh (2009)	Iran	Spring	Milk	Ewe	10	3	5–25	8/9
Rahimi, Shakerian, et al. (2009)	Iran	All	Milk	NR	236	213	NR	9/9
Rahimi, Karim, and Shakerian (2009)	Iran	All	Cheese	NR	88	47	NR	7/9
Ardic (2009)	Turkey	Spring	Milk	Ewe	90	80	17–232	6/9
Ardic et al. (2009)	Turkey	Spring	Cheese	NR	193	159	52–860	8/9
Aygun et al. (2009)	Turkey	Summer, autumn	Cheese	NR	120	72	16–1043	9/9
Var and Kabak (2009)	Turkey	NR	Milk, cheese	NR	60	46	10–388	6/9
Gündinç and Filazi (2009)	Turkey	Spring	Milk	NR	50	50	5–24 ng/kg	7/9
Arslan and EŞSİZ (2009)	Turkey	All	Milk	Cow	36	8	5.9–64.90	5/9
Herzallah (2009)	Jordan	Spring, winter	Milk	Cow, sheep and goat	60	5	NR	9/9
Dashti et al. (2009)	Kuwait	All	Cheese, milk	NR	322	313	2.42–452	8/9
Ghanem and Orfi (2009)	Syria	All	Milk	Cow, goat, sheep	118	100	6–765	7/9
MOHAMMADIAN et al. (2010)	Iran	All	Milk	NR	272	257	NR	8/9
Fallah (2010a)	Iran	Winter, summer	Milk, yogurt, cheese, ice cream	NR	267	195	13–1200	8/9
Fallah (2010b)	Iran	Autumn, spring	Milk	NR	225	151	5.6–528.5	8/9
Heshmati and Milani (2010)	Iran	All	Milk	NR	210	116	8–249	8/9
Mohamadi et al. (2010)	Iran	Summer	Milk, cheese	NR	160	160	NR	7/9
Rahimi et al. (2010)	Iran	Autumn	Milk	Cow, camel, sheep and goat	311	131	NR	8/9
Sani, Nikpooyan, and Moshiri (2010)	Iran	Spring	Milk	NR	196	19	19–126	9/9
Nemati et al. (2010)	Iran	All	Milk	NR	90	90	2.9–85.0	7/9
Filazi, Sinan, and Temamogullari (2010)	Turkey	NR	Cheese	Ewe	50	14	NR	7/9
Er et al. (2010)	Turkey	NR	Milk, cheese	NR	220	220	NR	8/9
Hampikyan et al. (2010)	Turkey	NR	Cheese	NR	80	41	52–2520	7/9
Atasever et al. (2010b)	Turkey	All	Cheese	NR	304	216	51–860	8/9
Aksoy et al. (2010)	Turkey	NR	Milk, cheese	cow	86	45	NR	8/9
Atasever et al. (2010a)	Turkey	All	Milk	NR	150	89	5–185	8/9
Mohamadi and Alizadeh (2010)	Iran	Summer	Milk, cheese	NR	160	160	NR	8/9
Rohani, Aminaee, and Kianfar (2011)	Iran	All	Milk	NR	72	36	NR	6/9
Sefidgar et al. (2011)	Iran	Winter	milk	NR	72	72	NR	7/9
Kamkar, Jahed, and Alavi (2011)	Iran	Autumn and winter	Milk	NR	122	122	4–112.4	9/9
Rahimi, Nilchian, and Behzadnia (2011)	Iran	All	Milk	NR	149	142	NR	8/9
Panahi et al. (2011)	Iran	Spring	Milk	Cow	100	100	1.3–68	9/9
Fallah et al. (2011)	Iran	All	Milk, cheese, yogurt, kashk and doogh	Cow, goat and sheep	682	316	13–394	8/9
Movassagh (2011)	Iran	Summer	Milk	NR	49	49	0–259	7/9
Maktabi et al. (2011)	Iran	All	Milk	NR	178	178	5–225	9/9
Buldu, Koc, and URAZ (2011)	Turkey	Winter	Milk	Cow	90	90	NR	7/9
Atasever, Atasever, and ÖZTURAN (2011)	Turkey	All	Yoghurt, doogh	NR	160	142	6–475	9/9
Kav, Col, and Tekinsen (2011)	Turkey	Winter	Cheese	Cow	127	36	70.61–770.97	8/9
Ertas et al. (2011)	Turkey	Spring	Milk, yoghurt, cheese	NR	210	135	1–369.5	7/9
El Khoury, Atoui, and Yaghi (2011)	Lebanon	NR	Milk, yogurt	NR	128	47	NR	6/9
Assem and Mohamad (2011)	Lebanon	Spring, summer	Milk	Cow, goat	63	45	2.63–126	6/9
Azizollahi Aliabadi et al. (2012)	Iran	Summer autumn	Cheese	NR	90	78	7.2–413	8/9
Sepehr et al. (2012)	Iran	NR	Milk, cheese	NR	220	220	0–260	8/9
Mohamadi Sani, Khezri, and Moradnia (2012)	Iran	Autumn	Milk	NR	42	41	NR	7/9
Issazadeh, Darsanaki, and Pahlaviani (2012)	Iran	Autumn	Yogurt	NR	60	59	6.2–87	8/9
Rahimi et al. (2012)	Iran	All	Milk, cheese	NR	121	61	11–209	8/9
Nilchian and Rahimi (2012)	Iran	NR	Yogurt, cheese, ice cream	Cow	120	104	11.4–505.7	7/9
Khoshnevis et al. (2012)	Iran	Autumn	Ice cream	NR	45	10	1.2–103	7/9
Tavakoli et al. (2012)	Iran	Summer, winter	Cheese	NR	50	30	40.9–374.0	9/9
Behfar et al. (2012)	Iran	Winter, spring	Milk	NR	100	100	0.45–9.76	6/9
Rahimi and Ameri (2012)	Iran	Autumn and spring	Milk	Cow, goat and sheep	150	70	8–115	9/9
Kocasari, Tasci, and Mor (2012)	Turkey	NR	Milk, cheese, yoghurt, ice cream	NR	225	165	5.5–600	9/9
Kabak and Ozbey (2012)	Turkey	Winter	Milk	Cow	40	8	NR	8/9
Omar (2012)	Jordan	Autumn, winter	Milk	NR	50	50	7.05–129.79	7/9
Elkak et al. (2012)	Lebanon	NR	Cheese	NR	111	75	5.61–315	8/9
Zuheir and Omar (2012)	Palestine	NR	Milk	NR	40	34	2–80	7/9
Behnamipour, Arast, and Mohammadian (2012)	Iran	Summer, winter	Milk, yogurt	NR	103	103	5–64	8/9
Tabari, Tabari, and Tabari (2013)	Iran	NR	Yogurt	NR	120	120	4.2–78.9	8/9
Yosef et al. (2013)	Saudi Arabia	Spring, summer	Milk	Cow	60	43	ND–455	5/9
S Aiad and Hs (2013)	Egypt	All	Milk, cheese and yoghurt	NR	150	67	6.20–133.2	8/9
Hashem and Abd‐Allah (2013)	Egypt	Summer	Milk, cheese	Buffalo, cow and camel	477	109.59	8–480	8/9
Kazemi, Azizollahi, and Mohammad (2013)	Iran	NR	Ice cream	NR	90	62	8.4–147.7	8/9
Sani and Nikpooyan (2013)	Iran	NR	Milk	NR	60	60	2–64	8/9
Tavakoli et al. (2013)	Iran	Summer, winter	Yogurt	NR	50	35	21.1–137.6	8/9
Ghaedi and Mohamadi Sani (2013)	Iran	NR	Milk	NR	34	30	5.33–248	7/9
Mohajeri et al. (2013)	Iran	Winter, spring	cheese	NR	82	39	70.5–309	8/9
Darsanaki et al. (2013)	Iran	Autumn, winter	Milk	NR	90	56	2.1–131	8/9
Riahi‐Zanjani and Balali‐Mood (2013)	Iran	Summer	Milk	NR	45	45	8.8–64	8/9
Khosravi et al. (2013)	Iran	All	Milk	NR	2160	2160	NR	9/9
Tosun and Ayyıldız (2013)	Turkey	All	Milk, cheese yogurt	NR	156	104	9–487	8/9
Moosavy et al. (2013)	Iran	Spring	Milk	NR	80	77	NR	7/9
Kamkar et al. (2014)	Iran	All	Milk	Cow and buffalo	120	90	3.6–422.7	9/9
Rahimi (2014)	Iran	NR	Ice cream, cheese and yogurt	NR	200	151	14.3–572.1	8/9
Rahimirad et al. (2014)	Iran	All	Milk, yogurt, cheese and cream	NR	300	225	NR	8/9
Khodadadi et al. (2014)	Iran	Winter	Cheese	NR	102	35	NR	9/9
Kara and Ince (2014)	Turkey	Summer, autumn and winter	Milk	buffalo and cow	250	34	<8	8/9
Bakırdere et al. (2014)	Turkey	NR	Cheese, milk	NR	243	131	5–2100	7/9
Sahindokuyucu Kocasari (2014)	Turkey	Spring, summer, autumn	Milk	NR	41	30	6.42–71.33	8/9
Temamogullari and Kanici (2014)	Turkey	Winter	Milk, cheese, yogurt	NR	150	150	0.82–130.89	9/9
Öztürk et al. (2014)	Cyprus	Autumn	Cheese	NR	128	30	0–16.66	9/9
Gul and Dervisoglu (2014)	Turkey	Autumn	Cheese	NR	147	144	15–3774	9/9
Golge (2014)	Turkey	All	Milk	NR	176	53	25–1101	6/9
Hassan and Kassaify (2014)	Lebanon	Autumn, spring	Milk, yogurt	Cow, goat, sheep	525	468	0–50	9/9
Christofidou et al. (2015)	Cyprus	NR	Milk, yogurt, cheese, ice cream	Cow, goat and sheep	1430	531	2–680	7/9
Mwanza et al. (2015)	Egypt	Spring, summer	Milk	NR	88	72	NR	9/9
El‐kest et al. (2015)	Egypt	NR	Milk, cheese yoghurt	NR	180	143	2.04–1622	9/9
Elsayed and Abd El‐Fatah (2015)	Egypt	NR	Yogurt, cheese milk	NR	65	21	0.2–47.1	7/9
Mahmoudi and Norian (2015)	Iran	Summer, winter	Milk	NR	288	163	NR	8/9
Fallah, Barani, and Nasiri (2015)	Iran	Summer, winter	Milk	Cow	254	204	11–321	9/9
Barikbin et al. (2015)	Iran	Winter	Milk, cheese	NR	161	91	0–339.61	9/9
Zanjani et al. (2015)	Iran	Summer	Milk	cow	45	45	6.3–23.3	8/9
Rouhi et al. (2015)	Iran	Winter, spring	Milk	NR	120	10	NR	9/9
Rezaei et al. (2015)	Iran	NR	Milk, cheese	NR	111	104	<5–350,500	9/9
Mason et al. (2015)	Iran	Spring, summer	Yogurt	NR	80	77	<5–91	7/9
Mohajeri et al. (2015)	Iran	Winter, spring	Milk	NR	87	84	0.14–68.17	8/9
Kocak et al. (2015)	Turkey	Autumn	Milk	NR	90	68	0.39–26.6	7/9
Sarica et al. (2015)	Turkey	Winter, spring	Milk, yogurt cheese	NR	70	62	7.3–107.2	6/9
Younis et al. (2016)	Egypt	NR	Milk, cheese	NR	30	20	10–200	7/9
Hashemi (2016)	Iran	All	Milk	NR	180	100	0–99.92	6/9
Dakhili, Shalibeik, and Ahmadi (2016)	Iran	Summer, winter	Milk	NR	70	65	11–296	8/9
Nikbakht et al. (2016)	Iran	Summer, autumn	Yogurt	NR	90	90	5–83	8/9
Ghajarbeygi et al. (2016)	Iran	All	Milk	Cow	60	34	6.25–127.87	7/9
Bahrami, Shahbazi, and Nikousefat (2016)	Iran	Winter, summer	Milk, cheese, yogurt, kashk, doogh	Cow, goat and sheep	358	175	5.7–272	9/9
Sohrabi and Gharahkoli (2016)	Iran	Summer, winter	Milk, yogurt, cheese	NR	77	61	3.3–96.1	7/9
Mashak et al. (2016)	Iran	NR	Milk	NR	30	30	<18–140	8/9
Fallah, Fazlollahi, and Emami (2016)	Iran	All	Milk	Cow, sheep, goat and camel	808	228	11–198	8/9
Taherabadi et al. (2016)	Iran	Winter	Milk	NR	117	117	2.4–231	7/9
Mohammadi et al. (2016)	Iran	All	Milk	NR	76	76	11.7–106.6	8/9
Tajik et al. (2016)	Iran	Winter	Milk	NR	360	280	5.4–510.8	9/9
Özgören and Kemal Seçkin (2016)	Turkey	Autumn	Cheese	NR	100	52	10.6–702	9/9
Sahin et al. (2016)	Turkey	All	Milk, yoghurt and doogh	Cow	205	22	23–100	8/9
Omar (2016)	Jordan	NR	Milk	Cow, sheep, goat and camel	130	130	9.71–216.78	7/9
Tahoun et al. (2017)	Egypt	NR	Milk, yogurt and cheese	NR	75	27	3.30–74.23	8/9
Koutamehr et al. (2017)	Iran	Spring, winter	Milk	NR	320	293	NR	8/9
Shahbazi, Nikousefat, and Karami (2017)	Iran	Winter summer	Cheese	NR	360	194	50.5–308.7	9/9
Shokri and Torabi (2017)	Iran	Autumn winter	Milk	Camel	70	52	5.19–150.17	6/9
Sharifzadeh et al. (2017)	Iran	All	Cheese	NR	100	52	50.2–424.4	9/9
Movassaghghazani and Ghorbiani (2017)	Iran	Autumn	Milk	Cow	48	48	NR	8/9
Babolhavaegi et al. (2018)	Iran	Summer	Milk	NR	586	25	NR	8/9
Yilidirim et al. (2018)	Turkey	All	Milk	Cow	154	154	0.08–10.11	8/9
ÖZTÜRK YILMAZ and Altinci (2018)	Turkey	NR	Milk, cheese	NR	77	43	2.46–47.81	6/9
Madali, Gulec, and Ayaz (2018)	Turkey	Spring Summer	Milk	Cow, goat	135	135	NR	9/9
Sakin et al. (2018)	Turkey	NR	Cheese	NR	30	16	NR	8/9
Hassan et al. (2018)	Qatar	NR	Milk, yogurt and cheese	NR	164	135	2.53–217.15	8/9
Shehab, El‐Leboudy, and Abo El‐Makarem (2019)	Egypt	NR	Cheese, yoghurt	NR	150	26	51–2030	8/9
Zakaria et al. (2019)	Egypt	Spring summer	milk	NR	90	37	53–207	8/9
F. Abdallah, Girgin, and Baydar (2019)	Egypt	Summer	Milk	Cow	20	20	20–90	8/9
Khaneghahi Abyaneh et al. (2019)	Iran	Winter	Milk	Cow	461	252	NR	8/9
Abyaneh et al. (2019)	Iran	Winter	Milk	NR	45	36	NR	7/9
Mahmoodi Maymand, Mazaheri, and Talebi Mehrdar (2019)	Iran	NR	Milk	NR	43	10	NR	7/9
Nejad, Heshmati, and Ghiasvand (2019)	Iran	All	Milk	NR	88	76	<5–120	7/9
Ansari, Pourjafar, and Christensen (2019)	Iran	Summer	Milk	NR	100	68	2–90	8/9
Moghaddam et al. (2019)	Iran	NR	Milk	NR	518	518	NR	8/9
Acaroz (2019)	Turkey	Autumn	Cheese	NR	80	51	25.30–201.27	6/9
Eker, Muratoglu, and Eser (2019)	Turkey	All	Milk, cheese	NR	360	174	5.14–213.5	7/9
Çetin et al. (2019)	Turkey	All	Milk	NR	24	24	17.83–202.15	5/9
El‐Tawab et al. (2020)	Egypt	Spring summer	Milk	NR	100	14	440–11,000	7/9
Ahmed et al. (2020)	Egypt	NR	Cheese	NR	140	63	NR	9/9
Ismaiel et al. (2020)	Egypt	All	Milk, cheese yogurt	NR	302	80	0–2230	9/9
Hajmohammadi et al. (2020)	Iran	Summer winter	Milk	Cow	60	59	<5000–240,000	8/9
Ahmadi (2020)	Iran	All	Milk	Cow	90	68	11.17–453.48	8/9
Khorshidi et al. (2022)	Iran	NR	Doogh, kashk	NR	110	82	5–85	8/9
Nejad, Heshmati, and Ghiasvand (2020)	Iran	NR	Cheese	NR	70	67	<5–287.18	7/9
Heshmati, Mozaffari Nejad, and Ghyasvand (2020)	Iran	All	Yogurt	NR	50	43	<5–98.65	8/9
Daou et al. (2020)	Lebanon	NR	Milk yogurt, doogh and cheese	NR	868	525	11–7350	8/9
El Tawila et al. (2020)	Egypt	Summer, autumn	Milk, liquid yogurt, cheese	NR	30	4	60–100	9/9
Murshed (2020)	Yemen	NR	milk, yogurt and cheese	NR	190	154	21–5955	7/9
Adam et al. (2021)	Egypt	Summer autumn	Cheese, yogurt	NR	200	80	<50–875.4	9/9
Jafari et al. (2021)	Iran	Autumn winter	Milk	NR	156	156	NR	9/9
Onmaz et al. (2021)	Turkey	All	Cheese	Cow, sheep, goat	120	8	NR	8/9
Esam et al. (2022)	Egypt	autumn winter	Cheese, milk	NR	105	99	<5–108.14	8/9
Taşçi, Erol, and Kocasari (2022)	Turkey	Autumn	Milk	Cow	82	48	5.06–50.63	8/9
Mohamadin, Rama, and Seboussi (2022)	United Arab Emirates	Winter, Spring	Milk	NR	42	4	2.8–7.4	8/9
Khalifa, Sallam, and Kasem (2023)	Egypt	Autumn	Milk	Camel	45	8	NR	7/9
Youssef et al. (2023)	Egypt	Autumn	Milk	Cow and buffalo	64	50	NR	7/9
Hamad et al. (2023)	Egypt	Autumn winter	Milk	Cow, camel, sheep and goat	100	87	3.70–490.30	9/9

Abbreviation: NR, not reported.

**FIGURE 2:**
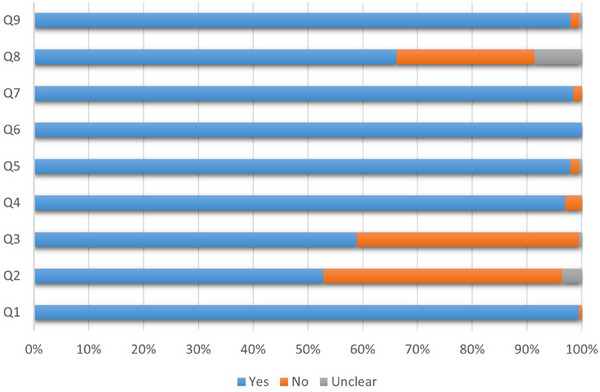
Quality of studies based on the type of questions in the JBI critical appraisal checklist. JBI, Joanna Briggs Institute.

A total of 5, 66 and 122 studies were classified as having low, fair and good quality, respectively. The quality of the 193 studies included in the analysis is presented in Table S3. Figure 2 shows the quality of studies based on the type of questions in the JBI checklist.

### 3.2 Publication Bias

On the basis of the results of the test (*I* ^2^: 98.5) and due to the heterogeneity of the selected studies, a random‐effects model was used to combine the studies. According to Begg's analysis, there was a significant publication bias considering the articles reporting AFM1 concentration (*p* < 0.001) or when papers assessing AFM1 occurrence rate and the percentage of AFM1 in samples were included in the analysis (*p* = 0.000). Furthermore, sensitivity analysis showed that none of the studies significantly influenced the effect size.

### 3.3 Meta‐Analysis Results

According to the study results, a total of 193 studies with 297,530 samples of dairy products were included in the meta‐analysis. The results showed that with 286,804 positive samples, 87% of dairy products were contaminated with AFM1. Subgroup analyses were performed on the basis of dairy product types, animal species, seasons, countries and various techniques for AFM1 detection to identify potential sources of heterogeneity. The pooled prevalence of AFM1 in dairy products, such as milk, yogurt, cheese, kashk, doogh and ice cream, was 87%, 95%CI (86.3%–87.8%); 68.9%, 95%CI (66.9%–70.8%); 63.6%, 95%CI (47.8%–79.5%); 62.9%, 95%CI (60.7%–65.1%); 55.6%, 95%CI (28.1%–83.2%) and 54%, 95%CI (27%–82%), respectively. The pooled prevalence of AFM1 in different types of milk, such as pasteurized, UHT, raw and traditional milk, was 99.5%, 95%CI (99.2%–99.7%); 91.3%, 95%CI (90%–92.6%); 73%, 95%CI (71.8%–74.2%) and 51%, 95%CI (49%–53.3%), respectively (Table 2). The pooled prevalence of AFM1 in different types of cheese, such as white, traditional, feta, processed and cheddar, was 95.7%, 95%CI (94.7%–96.7%); 86.7%, 95%CI (86.4%–87.1%); 66.5%, 95%CI (35.1%–97.9%); 32.3%, 95%CI (20.9%–43.7%) and 19.3%, 95%CI (12.6%–25.9%), respectively. The pooled prevalence of AFM1 in total yogurt and fruit yogurt (flavoured) was 68.9%, 95%CI (66.9%–70.8%) and 40%, 95%CI (33.2%–46.8%) (Table 2).

**TABLE 2:** Prevalence of aflatoxin M1 (AFM1) in dairy products based on subgroup analysis.

Dairy products	Prevalence of AFM1 (%)	[95% confidence intervals] (%)	Number of studies	*p* value	*I* ^2^ (%)
Milk					
Total milk	87	86.3–87.8	47	<0.0001	99.4
Pasteurized	99.5	99.2–99.7	22	<0.0001	99.6
UHT	91.3	90–92.6	15	<0.0001	97.6
Raw	73	71.8–74.2	52	<0.0001	98.8
Traditional	51	49–53.3	3	<0.0001	99.7
Cheese					
Total cheese	63.6	47.8–79.5	55	<0.0001	99.8
White	95.7	94.7–96.7	12	<0.0001	98.4
Traditional	86.7	86.4–87.1	9	<0.0001	96.2
Feta	66.5	35.1–97.9	2	<0.0001	96.5
Processed	32.3	20.9–43.7	2	<0.0001	99.4
Cheddar	19.3	12.6–25.9	2	<0.0001	99.6
Yogurt					
Total yogurt	68.9	66.9–70.8	35	<0.0001	99.6
Fruit yogurt (flavoured)	40	33.2–46.8	3	<0.0001	98.4
Doogh	55.6	28.1–83.2	6	<0.0001	94.4
Kashk	63.9	47.8–79.5	6	<0.0001	96.7
Ice cream	54	28.1–83.2	8	<0.0001	99.5
Animal					
Goat	84.5	77.8–91.3	3	<0.0001	99.4
Caw	78.2	76.3–80	20	<0.0001	98.2
Sheep	60.2	33–87.4	4	<0.0001	97.8
Camel	56	14.1–97.9	4	<0.0001	99.5
Season					
Spring	95.1	93.9–96.3	12	<0.0001	99.2
Winter	87	85.6–88.4	16	<0.0001	98.7
All‐season	81.9	81.9–92.4	92	<0.0001	92.9
Autumn	72	68.6–75.4	13	<0.0001	99.5
Summer	70.6	66.9–74.3	9	<0.0001	99.4
Detection method					
ELISA	88.1	81.4–89	140	<0.0001	99.5
HPLC	87.7	82.8–88.5	19	<0.0001	99.2
TLC	18.3	6.4–27.1	3	<0.0001	98.4
Immunoaffinity	13	9.3–21	2	<0.0001	97.9
RIDASCREEN	13.7	6.8–19.2	2	<0.0001	94.6
RT‐PCR	14	7.2–20.8	2	<0.0001	99.2
Country					
Iran	94.3	94–94.6	94	<0.0001	99.7
Jordan	80.4	75.6–85.1	3	<0.0001	98.5
Turkey	72.3	70.9–73.6	58	<0.0001	97.4
Kuwait	69.2	13.1–125.2	2	<0.0001	99.7
Lebanon	65.2	46.9–83.5	5	<0.0001	98.2
Syria	47.3	26.8–121.5	2	<0.0001	99.4
Egypt	46.7	27.9–65.4	19	<0.0001	99.6
Cyprus	25.1	6.5–43.8	3	<0.0001	99.7
UAE	17.7	2–37.4	2	<0.0001	96.8

Abbreviations: ELISA, enzyme‐linked immunosorbent assay; HPLC, high‐performance liquid chromatography; RT‐PCR, real‐time PCR; TLC, thin‐layer chromatography; UAE, United Arab Emirates; UHT, ultra‐high temperature.

**FIGURE 3:**
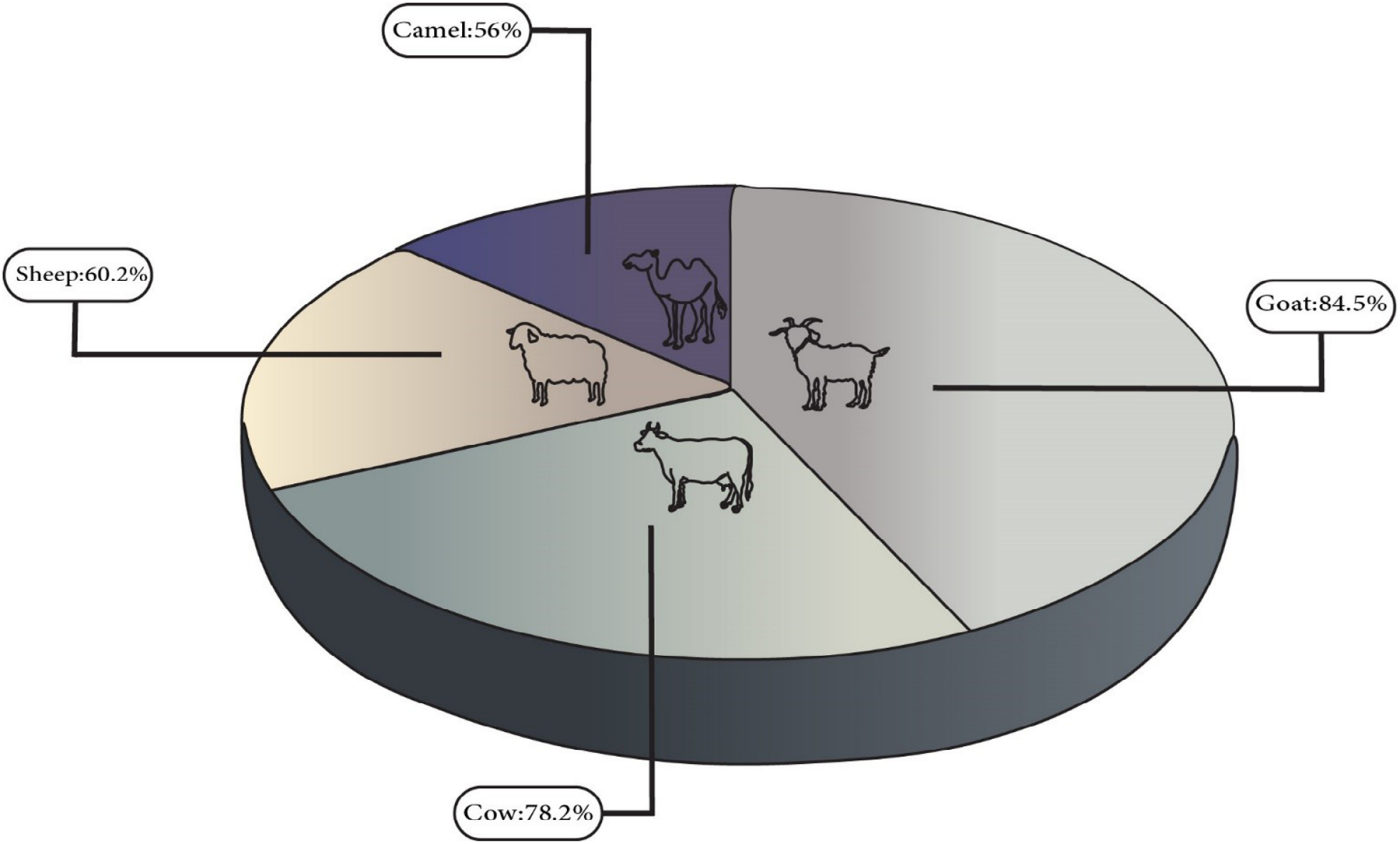
The pooled prevalence of AFM1 in different animals. AFM1, aflatoxin M1.

In the present study, a total of 60 studies with dairy product samples consisting of cow, sheep, goat and camel samples were analysed. According to the results, 63.74% of samples were contaminated with AFM1. The pooled prevalence of AFM1 in different types of animals, such as goat, cow, sheep and camel, was 84.5%, 95%CI (77.8%–91.3%); 78.2%, 95%CI (76.3%–80%); 60.2%, 95%CI (33%–87.4%); 56%, 95%CI (14.1%–97.9%) (Figure 3).

In the current study, 165 studies detected AFM1 in dairy products. According to the results, 88.1%, 87.7%, 18.3%, 14%, 13.7% and 11.3% of samples were detected with AFM1 by enzyme‐linked immunosorbent assay (ELISA), high‐performance liquid chromatography (HPLC), thin‐layer chromatography (TLC), immunoaffinity and real‐time PCR (RT‐PCR), respectively (Table 2).

## 4 Discussion

### 4.1 Prevalence of AFM1 in Dairy Products

Previous research has documented the occurrence of AFM1 in dairy products, with many studies focusing on milk samples. Raw milk, a popular dairy product consumed worldwide, has a limited shelf life, requiring careful handling to prolong its freshness and minimize the risk of disease transmission, particularly among vulnerable populations such as children and the elderly. Heat treatment is commonly employed during processing to ensure product safety and preserve its nutritional value. However, in some regions, the consumption of raw milk is culturally accepted, whereas heat‐treated milk is predominantly available in most markets globally (Abyaneh et al. 2020; Ghaffarian Bahraman et al. 2020; Mohammadi et al. 2021; Pour et al. 2020; Rahmani et al. 2018).

However, the most effective strategy for managing AF concentration in milk production involves implementing preventive measures to reduce animal exposure to this mycotoxin. Furthermore, factors such as climate conditions, geographical characteristics of the region, inadequate packaging, improper drying techniques and suboptimal practices in harvesting, handling, transportation and storage may create favourable conditions for fungal growth and subsequent mycotoxin contamination.

Despite the multifaceted nature of mycotoxin prevalence in food products across different countries, advancements in processing technologies, coupled with the implementation of stringent laws, policies and regulations, have significantly mitigated the risks associated with mycotoxin exposure in developed nations. Continuous monitoring and enforcement of these regulations have played a critical role in ensuring food safety and minimizing contamination levels, thereby protecting public health. Consequently, issues related to mycotoxin contamination are relatively rare in these regions. In contrast, populations in developing countries remain at a heightened risk of exposure to mycotoxins. This vulnerability can be attributed to several factors, including inadequate regulatory frameworks, limited access to modern agricultural practices and environmental conditions that favour fungal growth. Many individuals in these regions rely on subsistence farming and unregulated local markets for their food supply, which often lacks the rigorous safety standards seen in developed nations. The lack of resources and capacity within health systems further complicates efforts to monitor and address the impact of mycotoxins on human health. Research indicates that the adverse health effects associated with mycotoxin consumption have been documented for centuries. AF B1, one of the most studied mycotoxins, has been linked to severe health outcomes such as liver cancer. However, its broader implications—such as immune suppression and growth impairment—are only beginning to be understood within human populations. The intricate relationship between mycotoxin exposure and overall health burdens, particularly regarding infectious diseases, remains a significant concern that warrants further investigation. Thus, although, developed countries benefit from technological advancements and regulatory oversight that effectively reduce mycotoxin risks, developing nations face ongoing challenges that necessitate urgent attention to improve food safety and public health outcomes. Addressing these issues is essential for safeguarding vulnerable populations against the harmful effects of mycotoxins and enhancing overall food security (Cherkani‐Hassani, Mojemmi, and Mouane 2016). The fluctuation in AF levels found in dairy products can be linked to several factors. These include management practices throughout the production process, such as harvesting, storage and processing conditions, as well as environmental factors like humidity, temperature and rainfall. However, the primary contributors to variations in AF prevalence within dairy products are often the duration of storage and the hygienic conditions maintained in storage facilities (Kamika and Tekere 2016). These factors may create an environment conducive to the growth of mycotoxins (Kamika and Takoy 2011; Magan and Aldred 2007). At the retail level, AF contamination in dairy products can often be traced back to several systemic issues within transportation and food supply networks. These deficiencies may include inadequate drying facilities and limited storage capacity, which collectively contribute to the persistence of AFs in the food chain. To effectively mitigate AFM1 contamination in dairy products, it is essential to implement stringent controls on AFB1 contamination in grains. This can be accomplished through a multifaceted approach that includes the cultivation of crop varieties that are resistant to toxigenic fungi, minimizing physical damage from pests and employing effective crop rotation practices. Furthermore, it is crucial to recognize that grains deemed unfit for human consumption are equally unsuitable as feed for lactating animals. This highlights the interconnectedness of food safety across different sectors of the agricultural supply chain. Additionally, the drying process of feed prior to storage plays a pivotal role in preventing AF contamination. By creating unfavourable conditions for the growth of AF‐producing fungi—particularly concerning temperature and moisture levels—this step is vital in safeguarding both animal health and food safety (Beltrane and Junior 2005).

The utilization of ionizing radiation is a primary method employed for detoxifying AF. Conversely, chemical treatments, such as solvent extraction, various forms of chlorine (e.g., sodium hypochlorite and gas), oxidizing compounds like sodium bisulphate and hydrogen peroxide, as well as treatments involving ozone, ammonia, alkalis and acids, represent prevalent approaches to chemical detoxification. Recently, a significant focus has been on utilizing novel techniques, such as fermentation and integrating biological agents, namely, lactic acid bacteria (Campagnollo et al. 2016; Mousavi Khaneghah, Chaves, and Akbarirad 2017). Applying pasteurization and UHT procedures, which involve certain temperatures and durations, results in varying levels of AFM1 concentration in milk and milk‐derived dairy products (Campagnollo et al. 2016). AFM1 contamination in milk might result in the incidence of this mycotoxin in the final products. Insufficient research has been conducted to determine the effectiveness of heat in reducing AFM1 levels in dairy products. The pooled prevalence of AFM1 in the pasteurized, UHT, raw and traditional milk was 99.5, 95%CI (0.992–0.997); 91.3, 95%CI (0.900–0.926); 73, 95%CI (0.718–0.742); and 51, 95%CI (−0.230 to 1.251), respectively.

The findings of this study indicate that there were no significant differences in the estimated prevalence and concentration of AFM1 across various types of milk, including pasteurized, UHT, raw and traditional milk. This lack of distinction can be attributed to minor variations in the ranking of these milk types based on AFM1 prevalence and concentration, summarized as follows: pasteurized > UHT > raw > traditional (Table 2). Our results align with the previous research indicating that milk subjected to UHT treatment typically contains lower levels of AFM1 compared to pasteurized milk. For instance, Bakirci (2001) reported a 7.62% reduction in AFM1 concentration due to pasteurization. Similarly, Purchase et al. (1972) observed reductions of 32.5%, 45.5% and 63.6% in AFM1 levels when applying temperatures of 62°C for 30 min, 72°C for 45 s and 80°C for 45 s, respectively (Purchase et al. 1972). Furthermore, El‐Deeb et al. (1992) documented decreased AFM1 concentrations following both traditional and commercial sterilization heat treatments. The variations observed in these studies may stem from differences in processing temperatures and analytical methodologies employed across different research efforts. The sequential arrangement of milk product contamination with AFM1 (pasteurized > UHT > raw > traditional) can be explained by several factors: the potential presence of high levels of mycotoxin contamination in the pasteurized milk samples; the superior quality of raw or UHT milk compared to pasteurized options; inadequate heat treatment leading to reduced efficiency; or the use of lower pasteurization temperatures influenced by regional practices. Moreover, numerous studies have suggested that the conversion of various mycotoxins, particularly AFB1, into AFM1 during processing could contribute to the elevated levels observed in pasteurized milk (Berthiller et al. 2013; Khaneghah et al. 2021; Rychlik et al. 2014). The low contamination of raw milk and traditional with AFM1 in the present study can be related to its quick supply and absence of storage in tanks. In contrast, expansive reservoirs facilitate the transportation of milk to dairy factories, requiring the gathering of milk from multiple livestock or dairy farms to fill them. Meanwhile, dairy factories primarily get raw milk from industrial farms for pasteurization, sterilization and packaging. These farms often feed their animals with stockpiled animal feeds. These reasons might enhance the probability of AFM1 contamination in milk.

**FIGURE 4:**
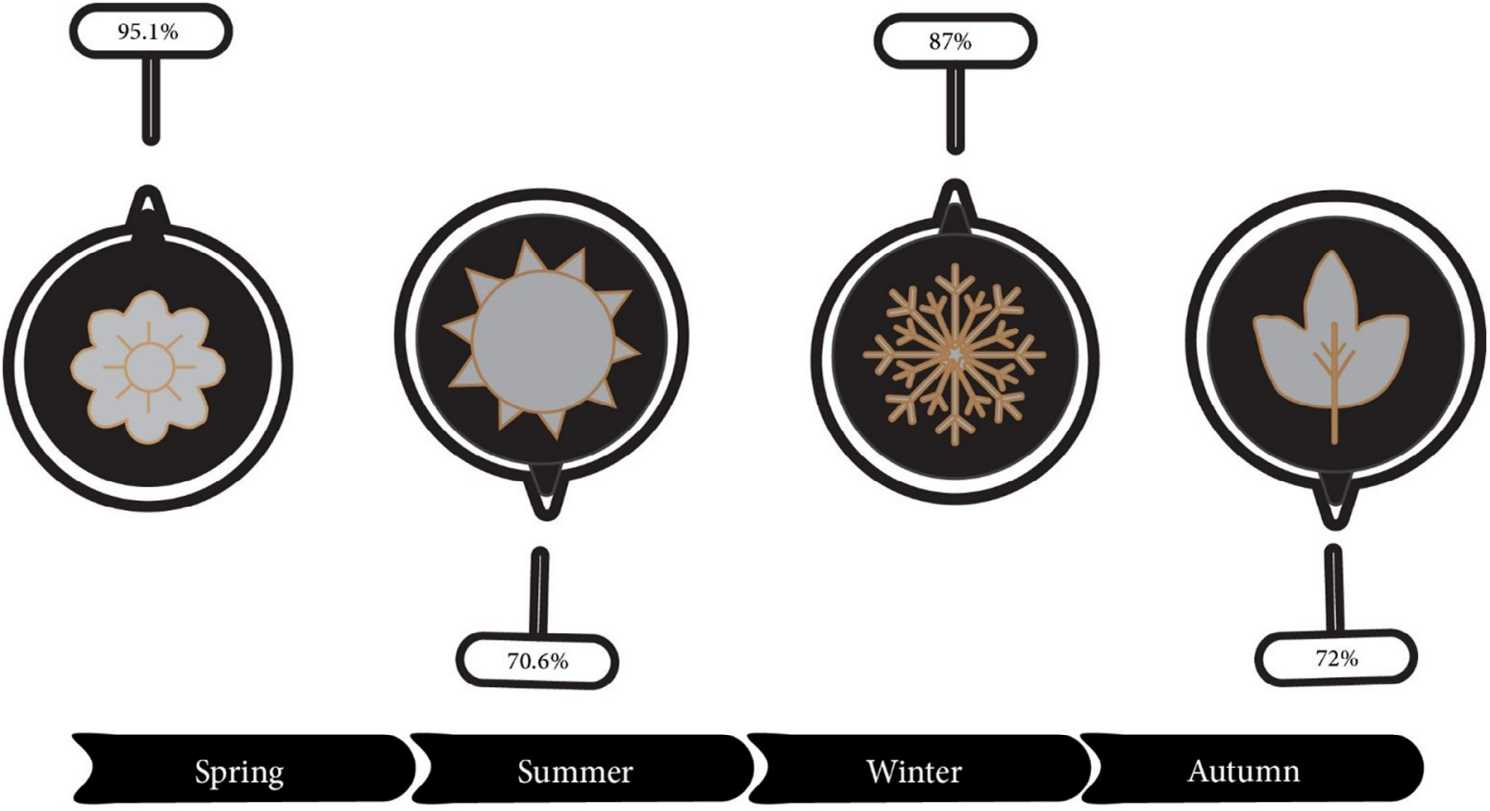
The pooled prevalence of AFM1 according to seasons. AFM1, aflatoxin M1.

The ranking of seasons in terms of the prevalence of AFM1 in dairy products is as follows: spring > winter > autumn > summer (Figure 4). Previous studies have demonstrated that various factors, such as food hygiene, geographical features, climatic conditions and toxin detection methods, can influence the level of AFM1 contamination in dairy products. Moreover, sampling time might influence the level and prevalence of AFM1 in dairy products (Ghaffarian Bahraman et al. 2020). Previous investigations have shown that the AFM1 concentration of dairy samples obtained in the winter is substantially higher than that of summer samples. Consistent with Fallah et al., our recent meta‐analysis reveals that the prevalence during winter and spring was higher than during summer. This could be attributed to the fact that lactating animals are typically fed stored forage and cereals during the winter, which are more prone to contamination with AFs than fresh feedstock (Fallah, Fazlollahi, and Emami 2016; Mohammadi et al. 2021).

The results of the present meta‐analysis showed the total prevalence of AFM1 in cheese samples. The pooled prevalence of AFM1 in the different types of cheese, such as white, traditional, feta, processed and cheddar, was 95.7, 95%CI (94.7, 96.7%); 86.7, 95%CI (86.4, 87.1%); 66.5, 95%CI (35.1, 97.9%); 32.3, 95%CI (20.9, 43.7%) and 19.3, 95%CI (12.6, 25.9%), respectively. Therefore, the ranking of cheese types according to the AFM1 prevalence can be summarized as follows: white > traditional > feta > processed > cheddar.

Although Khaneghah et al. reported on the overall contamination of AFM1 in cheese, their study lacked clarity regarding the sources of the samples and did not provide detailed information about the characteristics of the cheeses analysed. Furthermore, their findings revealed significant discrepancies when compared to previous research conducted in the West Asian region. For instance, Khaneghah et al. reported a mean AFM1 concentration of 14,000 ng/kg in hard cheeses; however, none of the studies we reviewed indicated such elevated levels (Khaneghah et al. 2021). The current meta‐analysis demonstrates that the occurrence rate of AFM1 in cheese was approximately 63.6%, indicating a relatively high concentration.

In summary, our findings underscore the critical need for stringent quality control measures throughout the dairy production chain to mitigate AFM1 contamination in both milk and cheese products. Understanding the dynamics of AF contamination not only contributes to food safety but also has broader implications for public health and regulatory policies aimed at ensuring consumer protection against mycotoxin exposure. By relating our findings to existing literature and highlighting these implications, we advocate for enhanced monitoring and improved practices within the dairy industry to safeguard public health effectively.

The primary source of AFM1 contamination in cheese is raw milk; however, inadequate quality control during the production process can exacerbate this issue (Campagnollo et al. 2016). Interestingly, our study found that the prevalence rate of AFM1 in milk (87%) was higher than that in cheese (63.6%). This contrasts with earlier studies, which demonstrated that AFM1 levels in cheese samples were significantly higher than those in corresponding milk samples that had been intentionally contaminated with AFM1 (Kamkar et al. 2008). One key factor contributing to the presence of AFM1 in cheese is its strong affinity for proteins such as casein (Campagnollo et al. 2016). This binding can lead to higher concentrations of AFM1 in cheese compared to milk. Moreover, multiple variables influence the rate of AFM1 contamination in cheese, including production conditions—such as temperature—as well as the chemical composition and physicochemical properties of the cheese itself, including texture type, pH and water activity (Gonçalves et al. 2020; Van Egmond 1989).

In summary, our findings underscore the critical need for stringent quality control measures throughout the dairy production chain to mitigate AFM1 contamination in both milk and cheese products. Understanding the dynamics of AF contamination not only contributes to food safety but also has broader implications for public health and regulatory policies aimed at ensuring consumer protection against mycotoxin exposure. By relating our findings to existing literature and highlighting these implications, we advocate for enhanced monitoring and improved practices within the dairy industry to safeguard public health effectively.

To effectively decrease AFM1 in milk and dairy products, minimizing animals’ contact with feedstock contaminated with AFB1 is advisable. This is because AFM1, a byproduct of AFB1, remains resistant to thermal treatments such as pasteurization and sterilization, making it difficult to eliminate (Ghaffarian Bahraman et al. 2020; Yousefi et al. 2018). Consistent with Khaneghah et al.’s findings, this meta‐analysis revealed that traditional fresh cheese samples had higher quantities of AFM1 compared to ripe samples (Khaneghah et al. 2021). One likely cause is that the protein content of traditional cheeses is higher than that of ripened products (Campagnollo et al. 2016). An investigation on Grana Padano cheese showed that the AFM1 concentration increased 4.5‐fold after 20 months of ripening (Manetta et al. 2009). It shows that the AFM1 concentration in white cheese had no significant changes after 3 months of storage (Oruc et al. 2006). An observational investigation of the cheddar cheese indicated a specific pattern in the AFM1 content over 40 weeks of ripening. According to this pattern, the toxin's concentration is low in the traditional fresh cheese, reaching its peak in the middle of the process and decreasing again at the end of the period (Brackett and Marth 1982). However, the AFM1 rate in different types of cheese, such as white, traditional and feta, compared to cheddar and processed cheese, was higher. Different levels of AFM1 degradation may occur in the different ripening processes.

### 4.2 Prevalence of AFM1 in Middle East Countries

Although the prevalence of AFM1 in dairy products among different countries is a multifactorial issue, the current meta‐analysis revealed that the Middle Eastern countries had a moderately higher prevalence of AFM1 in dairy products than other regions (Galvano, Galofaro, and Galvano 1996; Iqbal et al. 2015; Mulunda et al. 2013).

The prevalence of AFM1 in the dairy products of Iran, Jordan, Turkey, Kuwait, Lebanon, Syria, Egypt, Cyprus and the United Arab Emirates was 94.3, 95%CI (0.940–0.946); 80.4, 95%CI (0.756–0.851); 72.3, 95%CI (0.709–0.736); 69.2, 95%CI (0.131–1.252); 65.2, 95%CI (0.469–0.835); 47.3, 95%CI (−0.268 to 1.215); 46.7, 95%CI (0.279–0.654); 25.1, 95%CI (0.065–0.438) and 17.7, 95%CI (−0.020 to 0.374), respectively (Table 1).

**FIGURE 5:**
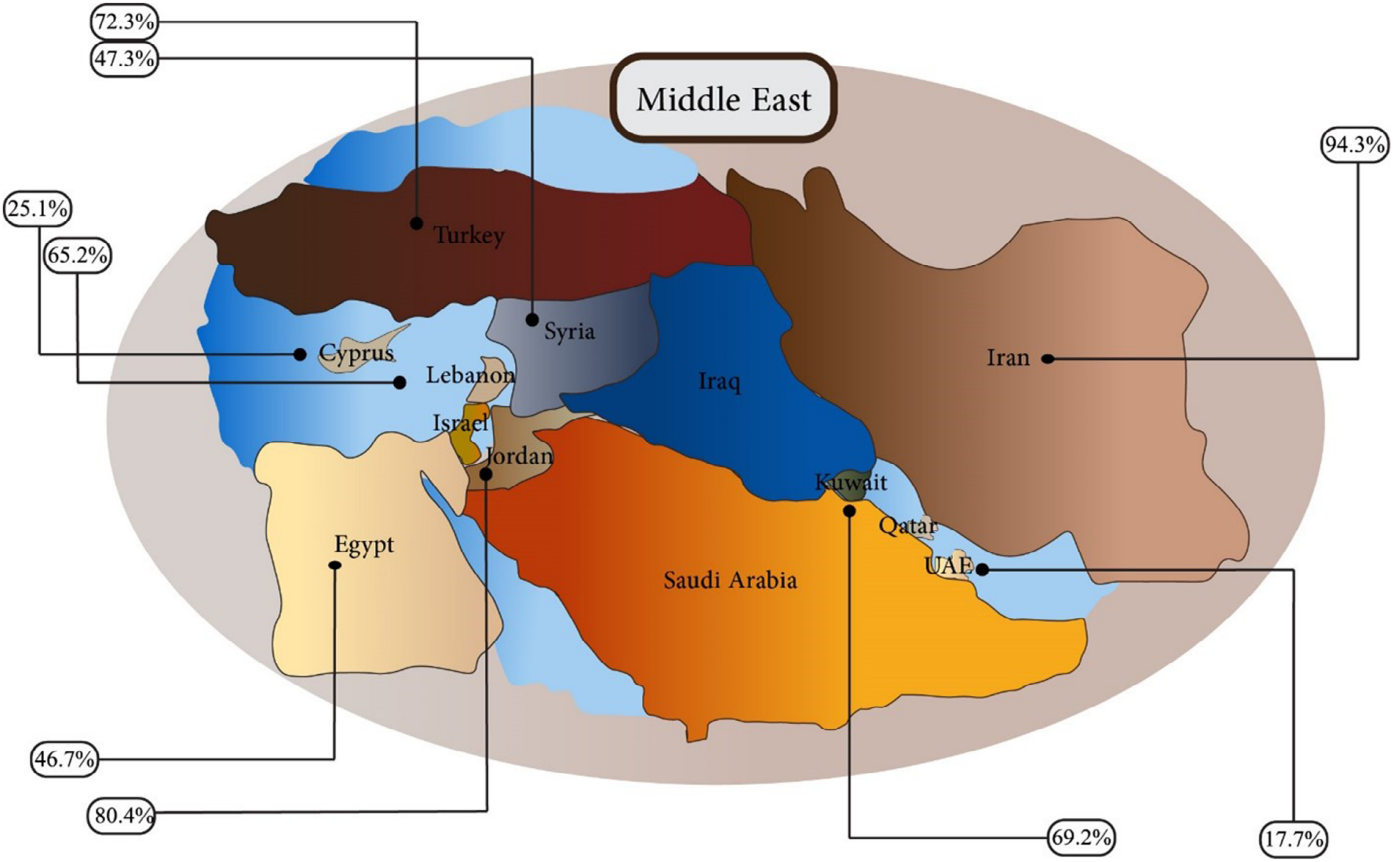
The pooled prevalence of AFM1 in Middle East countries. AFM1, aflatoxin M1.

The ranking of nations in terms of the prevalence of AFM1 in their dairy products is as follows: Iran > Jordan > Turkey > Kuwait > Lebanon > Syria > Egypt > Cyprus > the United Arab Emirates (Figure 5). The factors influencing the occurrence of AFM1 can be categorized as follows: sources (including quality and hygienic conditions), humidity levels and other economic, ecological and qualitative characteristics related to farm practices and management. In addition, the absence of rainfall also stimulates the growth of fungal species that produce mycotoxins, such as *Aspergillus*, leading to a significant increase in contamination rates (Tajkarimi et al. 2008; Tajkarimi et al. 2007). The Middle East region frequently faces drought conditions due to its high temperatures, which significantly impact agricultural productivity and increase the risk of mycotoxin contamination in milk and dairy products, particularly AFM1. Given these climatic challenges, one might expect consistently high levels of AFM1 across the region. However, our analysis reveals notable variations in AFM1 prevalence among the countries studied, despite their similar environmental conditions. These discrepancies can be attributed to several factors, including differences in agricultural practices, technological advancements and processing methods employed in each country. It is essential to recognize that each nation within the study demonstrates varying levels of technological development in its agricultural sector. Such differences can influence not only the efficiency of milk production but also the effectiveness of contamination control measures. For instance, countries with more advanced agricultural technologies may implement better irrigation practices and crop management strategies that can reduce mycotoxin levels in feed, thereby lowering the risk of AFM1 contamination in milk. Additionally, recent management strategies—such as Good Handling Practices (GHP), Good Agricultural Practices (GAP) and Hazard Analysis and Critical Control Points (HACCP)—have shown considerable effectiveness in minimizing AFM1 contamination risks within feeding and milk collection systems. The adoption of these practices is crucial for enhancing food safety and ensuring the quality of dairy products. The implications of our findings extend beyond individual countries; they highlight the importance of regional collaboration to share best practices and technological innovations aimed at reducing mycotoxin contamination. By fostering a collective approach to addressing these challenges, countries in the Middle East can improve their dairy production standards and enhance consumer safety. In conclusion, although environmental factors, such as drought, play a significant role in mycotoxin contamination, it is equally important to consider the impact of technological advancements and management practices on AFM1 levels in milk. Our study underscores the necessity for continuous improvement in agricultural practices and processing methods across the region to mitigate the risks associated with mycotoxin contamination effectively. This comprehensive understanding not only contributes to food safety but also supports public health initiatives aimed at reducing exposure to harmful contaminants in dairy products.

### 4.3 Prevalence of AFM1 in Animals

AFM1 was found in milk samples from 84.5% of goats, 78.2% of cows, 60.2% of sheep and 56% of camels. Hence, the total prevalence of AFM1 in dairy products from various animal species can be ranked as follows: goat > cow > sheep > camel. Nevertheless, variations in milk contamination levels across different animal species may arise from disparities in their digestive systems, processes of AFB1 absorption, types of feed, feeding methods and farm management techniques (Asi et al. 2012; Bahrami, Shahbazi, and Nikousefat 2016; Fallah, Fazlollahi, and Emami 2016; Hussain et al. 2010; Kos et al. 2014). Sheep, goat and camel herds are fed with stored grains or compound feedstuffs for a particular duration each year, and they have unlimited access to graze on pastureland for the remaining period. In contrast, cows are frequently provided with indoor feeding consisting of dry hay, cotton‐seed cake, maize or concentrated feedstuffs. These feedstuffs can be a significant source of AF contamination (Bahrami, Shahbazi, and Nikousefat 2016; Fallah, Fazlollahi, and Emami 2016; Rahimi and Ameri 2012). Indeed, it is commonly observed that AFM1 prevalence in milk samples from regions where cows have unrestricted access to grazing is typically lower compared to areas that employ closed systems relying on stored grains or compound feedstuff (Rohani, Aminaee, and Kianfar 2011). Hence, allowing animals to graze in open pastures is a highly efficient method for mitigating AFM1 contamination in bovine milk and several other animal species (Hussain et al. 2010; Mahmoudi and Norian 2015). Moreover, the concentration of AFM1 in the milk of various animals may be influenced by the AFM1 carryover ratio, which refers to the ratio of the steady concentration level of AFM1 in milk to the total amount of AFB1 consumed. The ratio varies among species and is mainly determined by lactation stage, milking time, udder health and productivity levels. The AFM1 carryover ratio typically ranges from 1% to 5%, with estimates of 0.35% to 3% in cows and 0.08% to 0.33% in sheep (Santini et al. 2013).

### 4.4 Detection Methods of AFM1

The rank order of the used methods for detecting AFM1 in dairy products: ELISA (140 studies) > HPLC (19 studies) > TLC (2 studies); immunoaffinity column assays (IAC) (2 studies) > RIDASCREEN (2 studies) (Table 1).

Immunological and chromatographic methods are commonly employed to quantify and detect AFM1 in dairy products. These techniques have several advantages, including reliability, rapidity, high sensitivity (especially for detecting low levels), selectivity and cost‐effectiveness. Chromatographic techniques, such as HPLC and TLC, confirm the initial results of rapid screening kits. Immunological techniques, such as ELISA, which depends on particular antibodies, are frequently employed to determine the prompt presence or absence of AFM1. Other methods include radioimmunoassay (RIA), sequential injection immunoassay and IAC. The results of the current meta‐analysis demonstrated that ELISA was considered the most prevalent screening method for detecting AFM1. However, if the contamination level of AFM1 is elevated, this method will be ineffective. Furthermore, the reliability of this approach is compromised by potential cross‐reaction interferences. Nevertheless, the researchers prefer a cost‐effective and uncomplicated approach. Lately, there have been advancements in developing detecting techniques such as the RIDASCREEN kits. The RIDASCREEN is based on an ELISA sandwich for rapidly quantifying proteins in various foods. According to studies, the most commonly used procedures for detecting and extracting AFM1 include IAC, SPE cartridges (C18), silica gel and liquid–liquid extraction. The current meta‐analysis showed that HPLC ranked second in terms of popularity. The common challenges associated with HPLC include the necessary time for sample preparation and running, which is crucial for ensuring the validity of results. In addition, toxic chemical solvents, such as pyridinium hydrobromide and trifluoroacetic acid, can be an essential risk factor to operators and the HPLC column, diminishing its lifetime. Nevertheless, the HPLC method surpasses other techniques in terms of precision, sensitivity and repeatability (Heshmati and Milani 2010). According to the TLC method, measuring significant contamination levels by AFM1 is not feasible. The Association of Analytical Communities (AOAC) has acknowledged TLC as a method for quantifying and identifying AFs at levels as high as 1 ng/g (Jalili and Scotter 2015; Kamkar 2005). Thus, TLC/HPLC was employed in several conducted studies (Sani and Nikpooyan 2013; Shundo and Sabino 2006). However, despite the advantages of simplicity, specificity and high sensitivity, the use of IAC for the quantitative determination of AFM1 has only been studied in two instances (Shundo and Sabino 2006; Tabari et al. 2011). The sensitivity and specificity of HPLC are higher than TLC and ELISA techniques (Bellio et al. 2016; Iqbal et al. 2014; Pirestani et al. 2011). Therefore, ELISA assays may yield a higher number of false‐positive results compared to HPLC (Iqbal et al. 2015). Bahrami et al. reported a significant rate of false‐positive results in ELISA assays. Out of the 113 milk samples (from cows, goats and sheep) that were found to contain AFM1 using the ELISA approach, only 80 samples were verified using HPLC analysis (Bahrami, Shahbazi, and Nikousefat 2016). Furthermore, Kim et al. demonstrated that ELISA assays better estimate AFM1‐positive samples than the HPLC approach (Kim et al. 2000).

The use of diverse analytical techniques, including ELISA, HPLC, LC–MS and TLC, has led to differences in the reported concentrations of AFM1 in dairy products in published surveys. This matter could potentially have a detrimental impact on the estimation of consumption and subsequent research assessing risk. To find accurate estimations of the intake of AFM1 through dairy product consumption, it is necessary to harmonize the obtained data.

### 4.5 Limitations, Strengths and Future Directions

One notable limitation of our study lies in the inherent constraints associated with conducting a systematic review. Despite our exhaustive search across reputable databases, potential language barriers and regional publication biases may have resulted in the exclusion of relevant studies. The decision to exclude non‐English publications introduces a language bias, and the exclusion of studies focusing on non‐dairy or non‐animal products may limit the generalizability of our findings beyond the scope of dairy products. These limitations could have influenced the overall prevalence estimates, potentially underrepresenting the true extent of AFM1 contamination in Middle Eastern dairy products. The exclusion of studies reporting on other product types might introduce a bias towards dairy‐centric results, impacting the comprehensiveness of our conclusions. Additionally, potential confounding variables, such as variations in climate, agricultural practices and storage conditions, may have influenced our results. Acknowledging these variables is crucial when interpreting prevalence rates and drawing conclusions about the factors contributing to AFM1 contamination.

A significant strength of our study lies in the extensive meta‐analysis of a large dataset encompassing diverse Middle Eastern countries, dairy product types and detection techniques. This comprehensive approach enhances the reliability and generalizability of our findings. Our research fills a critical gap in the existing literature by consolidating and synthesizing data from a broad range of studies. The detailed subgroup analyses, including variations by dairy product type, animal species and geographical location, enrich the understanding of AFM1 contamination dynamics. Furthermore, our study adheres to the PRISMA checklist and has been approved by PROSPERO.

Our findings carry significant implications for public health and food safety in the Middle East. The widespread prevalence of AFM1 in dairy products underscores the need for rigorous monitoring and regulatory measures to mitigate potential health risks associated with AF exposure. This study contributes to the growing body of knowledge on AFM1 contamination in dairy products, particularly in the Middle East. By synthesizing a large number of studies, our work provides a comprehensive overview, enabling a more nuanced understanding of the prevalence and distribution of this mycotoxin in the region.

To advance our understanding, future research should explore the specific factors contributing to observed variations in AFM1 contamination across different dairy product types, animal species and geographical regions. Investigating the effectiveness of different mitigation strategies and monitoring programmes would be instrumental in guiding regulatory interventions. Future research could benefit from standardized methodologies across studies, enabling more accurate comparisons. Incorporating unpublished data and addressing language barriers could enhance the comprehensiveness of systematic reviews in this field.

The practical applications of our research extend to regulatory bodies and food safety authorities in the Middle East. Implementing targeted monitoring programmes, setting stringent quality standards and educating stakeholders in the dairy industry can help mitigate AFM1 contamination, safeguarding public health. Our findings provide valuable insights for the dairy industry, enabling producers to implement effective quality control measures. Enhanced awareness of AFM1 prevalence in specific dairy products can guide industry practices to minimize contamination risks.

## 5 Conclusion

The findings of this study indicate a high prevalence of AFM1 in dairy products across Middle Eastern countries. Considering the significance of the milk group and its derivatives in consumption, it is imperative to implement specific measures to safeguard the food supply from AF moulds and ensure the quality of milk. Consequently, the application of reduction strategies becomes essential to decrease the AFM1 content in both milk and dairy products.

## Author Contributions

**B.A**.: conceptualization, methodology, formal analysis, project administration, writing–original draft preparation, writing–review and editing. **A.J**.: conceptualization, methodology, project administration, writing–original draft preparation, writing–review and editing, supervision. **K.K**.: data curation, writing–review and editing. **H.M**.: data curation, writing–review and editing. All authors have read and agreed to the published version of the manuscript.

## Ethics Statement

This study received the approval from the Abadan University of Medical Sciences Ethical Committee (IR.ABADANUMS. REC.1403.131, available at: https://ethics.research.ac.ir/IR.ABADANUMS.REC.1403.131). Ethical issues (including plagiarism, informed consent, misconduct, data fabrication and/or falsification, double publication and/or submission, redundancy, etc.) have been completely observed by the authors.

## Conflicts of Interest

The authors declare no conflicts of interest.

### Peer Review

The peer review history for this article is available at https://publons.com/publon/10.1002/vms3.70204.

## Supporting information



Supporting Information

## Data Availability

All relevant data are provided within the manuscript and supplementary file. Additionally, data analysed for this study are available upon request from the corresponding author.
