# Supplementary material for: A Comprehensive Systematic Review and Meta‐Analysis on the Prevalence of Aflatoxin M1 in Dairy Products in Selected Middle East Countries
Source: Vet Med Sci. 2025 Jan 22;11(1):e70204. doi: 10.1002/vms3.70204 (PMC11752159; doi:10.1002/vms3.70204)
Supplement: Supplementary file 1 — Supporting Information [file VMS3-11-e70204-s001.docx]

**A Comprehensive Systematic Review and Meta-Analysis on the Prevalence of Aflatoxin M1 in Dairy Products in Selected Middle East Countries**

**Contents**

[**Supplementary Table 1.** Search strategy to find potential eligible studies (August 2023) 2](#_Toc156584503)

[**Supplementary Table 2.** A summary of excluded articles after full text review. 4](#_Toc156584504)

[**Supplemental Table 3.** Quality of included studies by JBI critical appraisal checklist. 15](#_Toc156584505)

[**Supplemental References:** 23](#_Toc156584506)

| **Supplementary Table 1.** Search strategy to find potential eligible studies (August 2023) | |
| --- | --- |
| **Groups** | **Descriptors** |
| Mycotoxin | Aflatoxin M1 |
| Dairy type | (Milk OR yoghurt OR cheese OR ice-cream OR Cream OR curd OR kashk OR doogh OR colostrum) |

**PubMed**

Number of localized studies: 1,267

|  | **Descriptors** | **Number of studies reached** |
| --- | --- | --- |
| #1 | " Aflatoxin "[MeSH Terms] OR " Aflatoxin "[All Fields] OR " milk "[All Fields] OR " yoghurt "[All Fields] OR " cheese "[All Fields] OR "ice-cream "[All Fields] OR " Cream "[All Fields] OR " curd "[All Fields] OR " kashk "[All Fields] OR " doogh "[All Fields] OR " colostrum "[All Fields] | 1,267 |

**Web Of Science**

Number of localized studies: 2,399

|  | **Descriptors** | **Number of studies reached** |
| --- | --- | --- |
| #1 | TOPIC:( Aflatoxin) AND TOPIC:( milk) OR TOPIC:( yoghurt) OR TOPIC:( cheese) OR TOPIC:( ice cream) OR TOPIC:( Cream) OR TOPIC:( curd) OR TOPIC:( kashk) OR TOPIC:( doogh) OR TOPIC:( colostrum) | 2,399 |

**Scopus**

Number of localized studies: 2,538

|  | **Descriptors** | **Number of studies reached** |
| --- | --- | --- |
| #1 | (ALL FIELD (Aflatoxin)) AND (ALL FIELD (milk) OR ALL FIELD (yoghurt) ALL FIELD (cheese) OR ALL FIELD (ice-cream) OR ALL FIELD (Cream) OR ALL FIELD (curd) OR ALL FIELD (kashk) OR ALL FIELD (doogh) OR ALL FIELD (colostrum) ) | 2,538 |

| Supplementary Table 2. A summary of excluded articles after full text review. | |
| --- | --- |
| Author, Publication year (Ref.) | **Reason(s) for exclusion** |
| Ioannou‐Kakouri, 1995 (1) | Not enough data |
| Hassanin, 1993 (2) | Unrelated |
| Hamed, 2019 (3) | Unrelated / Plant-based Milk |
| Piekkola, 2012 (4) | Unrelated |
| Badria, 1996 (5) | Unrelated |
| Abdelhamid, 1990 (6) | Unrelated |
| El-Sawi, 1994 (7) | Unrelated |
| Megalla, 1982 (8) | Unrelated |
| Hafez, 1985 (9) | Colostrum drawn from cows postpartum |
| El-Sayed, 2002 (10) | Human breast milk |
| Kamel, 2017 (11) | Unrelated |
| Abdelmotilib, 2018 (12) | Unrelated |
| Wael, 2011 (13) | Formula and breast milk |
| Wael, 2011 (13) | Duplicate |
| Sebaei, 2020 (14) | Plant based |
| Gouda, 2019 (15) | Unrelated |
| Hassanin, 1994 (16) | Unrelated |
| Farag, 2020 (17) | Unrelated |
| El-Gohary, 2022 (18) | Investigation of aflatoxin in meats |
| Taherimaslak, 2014 (19) | Unrelated |
| Jahangiri–Dehaghani, 2020 (20) | Not enough data |
| Abyaneh, 2020 (21) | Meta-analysis study |
| Jalili, 2015 (22) | Review |
| Bodbodak, 2018 (23) | Unrelated |
| Hajmohammadi, 2021 (24) | Unrelated |
| Foroughi, 2018 (25) | Unrelated |
| Mahmoudi, 2014 (26) | Non-English |
| Kamkar, 2014 (27) | Review |
| Rezaei, 2015 (28) | Review |
| Riazipour, 2010 (29) | Non-English |
| Yahyaraeyat, 2017 (30) | Non-English |
| Kamkar, 2008 (31) | Duplicate (69) |
| Kamkar, 2011 (32) | Duplicate (72) |
| Pirestani, 2011 (33) | Unrelated |
| Mohammadian, 2010 (34) | Duplicate (68) |
| Rahimi, 2011 (35) | Duplicate (76) |
| Jafarian-Dehkordi, 2013 (36) | Human breast milk |
| Mahmoudi, 2015 (37) | Duplicate (59) |
| Mahmoudi, 2015 (37) | Duplicate (59) |
| Tabari, 2011 (38) | Unrelated |
| Bayat, 2021 (39) | Not interested outcome |
| Naeimipour, 2018 (40) | Unrelated |
| Manoochehri, 2015 (41) | Powder milk |
| Hashemi, 2014 (42) | Not interested outcome |
| Khorshidi, 2022 (43) | Duplicate |
| Hajmohammadi, 2020 (44) | Duplicate (53) |
| Heshmati, 2019 (45) | Herbal product |
| Naghshbandi, 2023 (46) | Powder milk |
| Mohammadi, 2021 (47) | Not interested outcome |
| Hashemi, 2019 (48) | Unrelated |
| Khoshpey, 2011 (49) | Review |
| Sefidgar, 2011 (50) | Duplicate (71) |
| Ataee, 2012 (51) | Short Communication |
| Ghiasian, 2012 (52) | Breast Milk |
| Mohammadi, 2008 (53) | Not interested outcome |
| Mohammadi, 2009 (54) | Duplicate (126) |
| Abnous, 2021 (55) | Unrelated |
| Kamkar, 2008 (56) | Did not report the number of samples |
| Fallah, 2009 (57) | Duplicate (104) |
| Gandomi, 2009 (58) | Unrelated |
| Hooshfar, 2020 (59) | Formula Milk |
| Sadeghi, 2009 (60) | Human breast milk |
| Heshmati, 2010 (61) | Duplicate (109) |
| Fallah, 2010 (62) | Duplicate (105) |
| Fallah, 2011 (63) | Duplicate (106) |
| Afshar, 2013 (64) | Human breast milk |
| Bahrami, 2016 (65) | Duplicate (95) |
| Sarlak, 2017 (66) | Unrelated |
| Sarmast, 2021 (67) | Systematic review and meta-analysis |
| Hashemi, 2014 (68) | Unrelated |
| Amoli-Diva, 2015 (69) | Not interested outcome |
| Fakhri, 2019 (70) | Human breast milk |
| Danesh, 2018 (71) | Review |
| Karimi Dehcheshmeh, 2021 (72) | Formula |
| Zakaria, 2019 (73) | Duplicate (28) |
| Karimi, 2022 (74) | Formula |
| Movassagh, 2013 (75) | Non-English |
| Khoori, 2020 (76) | Unrelated |
| Gholipour, 2012 (77) | Non-English |
| Moeinian, 2014 (78) | Non-English |
| Ghariby, 2017 (79) | Non-English |
| Najafian, 2015 (80) | Non-English |
| Gholamalian, 2022 (81) | Unrelated |
| Amiridumari, 2013 (82) | unrelated |
| Kamkar, 2006 (83) | Duplicate (142) |
| Moeinian, 2013 (84) | Non-English |
| Ahmadi, 2021 (85) | Non-English |
| Bahrami, 2016 (86) | Unrelated |
| Oveisi, 2007 (87) | Duplicate (143) |
| Sheini, 2020 (88) | Unrelated |
| Mahdavi, 2010 (89) | Human breast milk |
| Riahi-Zanjani, 2013 (90) | Duplicate (194) |
| Mohammadi, 2016 (91) | Duplicate (196) |
| Khosravi, 2013 (92) | Duplicate (199) |
| Riahi-Zanjani, 2019 (93) | Unrelated |
| Hassanpour, 2019 (94) | Unrelated |
| Pardakhti, 2019 (95) | Not interested outcome |
| Beitollahi, 2020 (96) | Review |
| Mason, 2016 (97) | Not interested outcome |
| Tajkarimi, 2007 (98) | Duplicate (157) |
| Tajkarimi, 2008 (99) | Duplicate (144) |
| azizi, 2014 (100) | Non-English |
| Moghaddam, 2019 (101) | Duplicate (218) |
| Pour, 2020 (102) | Meta-analysis study |
| Al-Sawaf, 2012 (103) | Milk powder |
| Mayer, 1969 (104) | Not interested outcome |
| Britzi, 2013 (105) | Unrelated |
| Britzi, 2013 (105) | Duplicate (224) |
| Deveci, 2006(106) | Unknown number of samples |
| Temamogullari, 2014 (107) | Duplicate (333) |
| Er, 2014 (108) | Sample: infant food sample |
| Elmali, 2008 (109) | Sample: milk powder |
| Oruc, 2006 (110) | Unrelated (adding AFM1!) |
| Kabak, 2008 (111) | Not detecting AFM1 |
| Gürbay, 2006 (112) | Duplicate (275) |
| Sarimehmetoğlu, 2004 (113) | Not detecting AFM1 |
| Baydar, 2007 (114) | Sample: infant formula |
| Bakirdere, 2014 (115) | Duplicate (293) |
| Aycicek, 2005 (116) | Duplicate (295) |
| Kivanc, 1992 (117) | Unrelated to AFM1 |
| Er Demirhan, 2021 (118) | Sample: Cereal-Based Baby Foods |
| Tasci, 2011 (119) | Unrelated to AFM1 |
| Guzel‐Seydim, 2006 (120) | Unrelated to AFM1 |
| Var, 2009 (121) | Duplicate (303) |
| Gürbay, 2010 (122) | Sample: breast milk |
| Aycicek, 2005 (116) | Duplicate (295) |
| Yaroglu, 2005 (123) | Duplicate (308) |
| Tekinşen, 2008(124) | Duplicate (265) |
| Ardic, 2009 (125) | Duplicate (291) |
| Kabak, 2012(126) | Sample: formula |
| Colak, 2007(127) | Review Article |
| Virdis, 2008 (128) | Not middle east (Italy) |
| Atasever, 2011 (129) | Duplicate (288) |
| Kılıç Altun, 2017 (130) | Sample: breast milk |
| Sahindokuyucu Kocasari, 2014 (131) | Duplicate (329) |
| Nilüfer, 2002 (132) | Sample: Tahini |
| Temamogullari, 2014 (107) | Duplicate (333) |
| Aygun, 2009 (133) | Duplicate (292) |
| Torlak, 2013 (134) | Sample: Tahini |
| Atatsever, 2021 (135) | Not middle east (Kyrgyz Republic) |
| Aksoy, 2016 (136) | Sample: Butter |
| Sanli, 2012 (137) | Unknown number of samples |
| Atasever, 2010 (138) | Sample: Butter |
| Gul, 2014 (139) | Duplicate (348) |
| Dinckaya, 2011 (140) | Not detecting AFM1 |
| Kabak, 2021 (141) | Not detecting AFM1 |
| Atasever, 2014 (142) | Sample: Breast milk |
| Kivanc, 1990 (143) | Not detecting AFM1 |
| Sevim, 2019 (144) | Not detecting AFM1 |
| Ayar, 2007 (145) | Duplicate (357) |
| Akgönüllü, 2021 (146) | Unrelated |
| Kav, 2011 (147) | Duplicate (316) |
| Colak, 2007 (148) | Unrelated (Adding AFM1) |
| Oruc, 2007 (149) | Unrelated (Adding AFM1) |
| Deveci, 2007 (150) | Unknown number of samples |
| Awaisheh, 2019 (151) | Sample: Infant milk formula |
| Omar, 2012 (152) | Duplicate (371) |
| Bani Ismail, 2020 (153) | Sample: Feed samples |
| Dashti, 2009 (154) | Duplicate (377) |
| Assaf, 2019 (155) | Unrelated (About elimination of AFM1) |
| Elkak, 2012 (156) | Duplicate (381) |
| Hassan, 2014 (157) | Duplicate (382) |
| Elaridi, 2019 (158) | Sample: baby formula |
| Daou, 2022 (159) | Sample: Infant formula |
| Al Zuheir, 2012 (160) | Duplicate (390) |
| Al Jabir, 2019 (161) | Not interested outcome (not detecting AFM1) |
| UI Hassan, 2018 (162) | Not interested exposure (Cereal-based baby food) |
| UI Hassan, 2018 (162) | Duplicate (394) |
| Chrouda, 2022 (163) | Unrelated |
| Aly, 2008 (164) | not detecting AFM1 |
| Elsanhoty, 2014 (165) | Unrelated (Detoxification of AFM1) |
| Hashem, 2013 (166) | Duplicate (29) |
| Abdulrazzaq, 2003 (167) | Sample: Cereal-based baby food |
| Mohamadin, 2022 (168) | Duplicate (403) |
| Al-Zenki, 2007 (169) | not interested outcome |
| Tajkarimi, 2007 (98) | not interested outcome |

| Supplemental Table 3. Quality of included studies by JBI critical appraisal checklist. | | | | | | | | | | |
| --- | --- | --- | --- | --- | --- | --- | --- | --- | --- | --- |
| Study, year (Ref) | Q1 | Q2 | Q3 | Q4 | Q5 | Q6 | Q7 | Q8 | Q9 | Overall quality |
| Saad, 1989 (170) | Y | N | N | Y | Y | Y | Y | N | Y | Fair |
| Haydar, 1990 (171) | Y | Y | N | Y | Y | Y | Y | N | Y | Fair |
| Ioannou-Kakouri, 1999 (172) | Y | Y | Y | Y | Y | Y | Y | Y | Y | Good |
| Bakirci, 2001 (173) | Y | Y | Y | Y | Y | Y | Y | Y | Y | Good |
| Ivastava Sr, 2001 (174) | Y | Y | N | Y | Y | Y | Y | N | Y | Fair |
| Hismiogullari, 2003 (175) | Y | N | N | N | Y | Y | Y | Y | Y | Fair |
| Gürses, 2004, (176) | Y | Y | N | Y | Y | Y | Y | N | Y | Fair |
| Sarimehmetoglu, 2004 (177) | Y | Y | Y | Y | Y | Y | Y | U | Y | Good |
| Kamkar, 2005 (178) | Y | Y | Y | Y | Y | Y | Y | Y | Y | Good |
| Çelik, 2005 (179) | Y | N | N | Y | Y | Y | Y | U | Y | Fair |
| Kamber, 2005 (180) | Y | Y | N | Y | Y | Y | Y | U | Y | Fair |
| Aycicek, 2005 (116) | Y | Y | Y | Y | Y | Y | Y | N | Y | Good |
| Yaroglu, 2005 (123) | Y | N | Y | Y | Y | Y | Y | Y | Y | Good |
| Tekinşen, 2005 (181) | Y | Y | Y | Y | Y | Y | N | Y | Y | Fair |
| Alborzi, 2006 (182) | Y | N | Y | Y | Y | Y | Y | Y | Y | Good |
| Kamkar, 2006 (83) | Y | Y | N | Y | Y | Y | Y | N | Y | Fair |
| Akkaya, 2006 (183) | Y | Y | Y | Y | Y | Y | Y | U | Y | Good |
| Gürbay, 2006 (112) | Y | Y | N | Y | Y | Y | Y | U | Y | Fair |
| Bașkaya, 2006 (184) | Y | N | Y | Y | Y | Y | Y | U | Y | Fair |
| Unusan, 2006 (185) | Y | N | Y | Y | Y | Y | Y | N | Y | Fair |
| Gürbay, 2006 (186) | Y | N | N | Y | Y | Y | Y | N | Y | Fair |
| Colak, 2006, (187) | Y | N | N | N | Y | Y | Y | N | Y | Poor |
| Tajik, 2007 (188) | Y | Y | Y | Y | Y | Y | Y | U | Y | Good |
| Karimi, 2007 (189) | Y | N | Y | Y | Y | Y | Y | Y | Y | Good |
| Ghiasian, 2007 (190) | Y | N | Y | Y | Y | Y | Y | Y | Y | Good |
| Oveisi, 2007 (87) | Y | N | Y | Y | Y | Y | Y | N | Y | Fair |
| Tajkarimi, 2008 (99) | Y | N | Y | Y | Y | Y | Y | Y | Y | Good |
| Oezdemir, 2007 (191) | Y | Y | Y | Y | Y | Y | Y | Y | Y | Good |
| Ayar, 2007 (145) | Y | N | N | Y | Y | Y | N | Y | Y | Poor |
| Kamkar, 2008 (31) | Y | Y | N | Y | Y | Y | Y | Y | Y | Good |
| Tekinşen, 2008 (124) | Y | Y | N | Y | Y | Y | Y | Y | Y | Good |
| Yapar, 2008 (192) | Y | N | Y | Y | Y | Y | Y | U | Y | Fair |
| Tekinşen, 2008 (193) | Y | Y | Y | Y | Y | Y | Y | Y | Y | Good |
| Akkaya, 2009 (194) | Y | N | Y | Y | Y | Y | Y | N | Y | Fair |
| Motawee, 2009 (195) | Y | Y | Y | Y | Y | Y | Y | N | Y | Good |
| Fallah, 2009 (57) | Y | Y | Y | Y | Y | Y | Y | N | Y | Good |
| Ghazani, 2009 (196) | Y | Y | N | Y | Y | Y | Y | Y | Y | Good |
| Movassagh, 2009 (197) | Y | Y | N | Y | Y | Y | Y | Y | Y | Good |
| Rahimi, 2009 (198) | Y | Y | Y | Y | Y | Y | Y | Y | Y | Good |
| Rahimi, 2009 (199) | Y | Y | N | N | Y | Y | Y | Y | Y | Fair |
| Ardic, 2009 (200) | Y | N | N | Y | Y | Y | Y | N | Y | Fair |
| Ardic, 2009 (125) | Y | Y | Y | Y | Y | Y | Y | U | Y | Good |
| Aygun, 2009 (133) | Y | Y | Y | Y | Y | Y | Y | Y | Y | Good |
| Var, 2009 (121) | Y | N | N | Y | Y | Y | Y | N | Y | Fair |
| Gündinç, 2009 (201) | Y | Y | N | Y | Y | Y | Y | N | Y | Fair |
| Arslan, 2009 (202) | Y | N | N | Y | U | Y | Y | Y | U | Poor |
| Herzallah, 2009 (203) | Y | Y | Y | Y | Y | Y | Y | Y | Y | Good |
| Dashti, 2009 (154) | Y | Y | Y | Y | Y | Y | Y | N | Y | Good |
| Ghanem, 2009 (204) | Y | N | Y | Y | Y | Y | Y | N | Y | Fair |
| Mohammadian, 2010 (34) | Y | N | Y | Y | Y | Y | Y | Y | Y | Good |
| Fallah, 2010 (62) | Y | N | Y | Y | Y | Y | Y | Y | Y | Good |
| Fallah, 2010 (205) | Y | Y | Y | Y | Y | Y | Y | N | Y | Good |
| Heshmati, 2010 (61) | Y | N | Y | Y | Y | Y | Y | Y | Y | Good |
| Mohamadi, 2010 (206) | Y | N | Y | Y | Y | Y | Y | N | Y | Fair |
| Rahimi, 2010 (207) | Y | N | Y | Y | Y | Y | Y | Y | Y | Good |
| Sani, 2010 (208) | Y | Y | Y | Y | Y | Y | Y | Y | Y | Good |
| Nemati, 2010 (209) | Y | N | N | Y | Y | Y | Y | Y | Y | Fair |
| Filazi, 2010 (210) | Y | N | Y | Y | Y | Y | Y | N | Y | Fair |
| Er, 2010 (211) | Y | N | Y | Y | Y | Y | Y | Y | Y | Good |
| Hampikyan, 2010 (212) | Y | Y | N | Y | Y | Y | Y | U | Y | Fair |
| Atasever, 201 (213) | Y | N | Y | Y | Y | Y | Y | Y | Y | Good |
| Aksoy, 2010 (214) | Y | Y | Y | Y | Y | Y | Y | N | Y | Good |
| Atasever, 2010 (215) | Y | Y | Y | Y | Y | Y | Y | U | Y | Good |
| Mohamadi, 2010 (216) | Y | N | Y | Y | Y | Y | Y | Y | Y | Good |
| Rohani, 2011 (217) | Y | N | N | Y | Y | Y | Y | N | Y | Fair |
| Sefidgar, 2011 (50) | Y | N | N | Y | Y | Y | Y | Y | Y | Fair |
| Kamkar, 2011 (32) | Y | Y | Y | Y | Y | Y | Y | Y | Y | Good |
| Rahimi, 2011 (35) | Y | N | Y | Y | Y | Y | Y | Y | Y | Good |
| Panahi, 2011 (218) | Y | Y | Y | Y | Y | Y | Y | Y | Y | Good |
| Fallah, 2011 (63) | Y | N | Y | Y | Y | Y | Y | Y | Y | Good |
| Movassagh, 2011 (219) | Y | Y | N | Y | Y | Y | Y | N | Y | Fair |
| Maktabi, 2011 (220) | Y | Y | Y | Y | Y | Y | Y | Y | Y | Good |
| Buldu, 2011 (221) | Y | N | N | Y | Y | Y | Y | Y | Y | Fair |
| Atasever, 2011 (129) | Y | Y | Y | Y | Y | Y | Y | Y | Y | Good |
| Kav, 2011 (147) | Y | N | Y | Y | Y | Y | Y | Y | Y | Good |
| Ertas, 2011 (222) | Y | N | Y | Y | Y | Y | Y | U | Y | Fair |
| El Khoury, 2011 (223) | Y | N | Y | N | Y | Y | Y | U | Y | Fair |
| Assem, 2011 (224) | Y | N | N | Y | Y | Y | Y | N | Y | Fair |
| Azizollahi Aliabadi, 2012 (225) | Y | Y | N | Y | Y | Y | Y | Y | Y | Good |
| Sepehr, 2012 (226) | Y | N | Y | Y | Y | Y | Y | Y | Y | Good |
| Mohamadi Sani, 2012 (227) | Y | N | N | Y | Y | Y | Y | Y | Y | Fair |
| Issazadeh, 2012 (228) | Y | Y | N | Y | Y | Y | Y | Y | Y | Good |
| Rahimi, 2012 (229) | Y | N | Y | Y | Y | Y | Y | Y | Y | Good |
| Nilchian, 2012 (230) | Y | N | Y | Y | Y | Y | Y | N | Y | Fair |
| Khoshnevis, 2012 (231) | Y | Y | N | Y | Y | Y | Y | N | Y | Fair |
| Tavakoli, 2012 (232) | Y | Y | Y | Y | Y | Y | Y | Y | Y | Good |
| Behfar, 2012 (233) | N | N | Y | Y | Y | Y | Y | N | Y | Fair |
| Rahimi, 2012 (234) | Y | Y | Y | Y | Y | Y | Y | Y | Y | Good |
| Kocasari, 2012 (235) | Y | Y | Y | Y | Y | Y | Y | Y | Y | Good |
| Kabak, 2012 (236) | Y | N | Y | Y | Y | Y | Y | Y | Y | Good |
| Omar, 2012 (152) | Y | N | Y | Y | Y | Y | Y | N | Y | Fair |
| Elkak, 2012 (156) | Y | Y | Y | Y | Y | Y | Y | U | Y | Good |
| Zuheir, 2012 (237) | Y | N | N | Y | Y | Y | Y | Y | Y | Fair |
| Behnamipour, 2012 (238) | Y | Y | Y | Y | Y | Y | Y | N | Y | Good |
| Tabari, 2013 (239) | Y | U | Y | Y | Y | Y | Y | Y | Y | Good |
| Yosef, 2013 (240) | Y | U | N | Y | N | Y | Y | Y | N | Poor |
| Aiad, 2013 (241) | Y | Y | Y | Y | Y | Y | Y | N | Y | Good |
| Hashem, 2013 (166) | Y | N | Y | Y | Y | Y | Y | Y | Y | Fair |
| Kazemi, 2013 (242) | Y | Y | N | Y | Y | Y | Y | Y | Y | Good |
| Sani, 2013 (243) | Y | Y | N | Y | Y | Y | Y | Y | Y | Good |
| Tavakoli, 2013 (244) | Y | Y | N | Y | Y | Y | Y | Y | Y | Good |
| Ghaedi, 2013 (245) | Y | N | N | Y | Y | Y | Y | Y | Y | Fair |
| Mohajeri, 2013 (246) | Y | Y | N | Y | Y | Y | Y | Y | Y | Good |
| Darsanaki, 2013 (247) | Y | Y | N | Y | Y | Y | Y | Y | Y | Good |
| Riahi-Zanjani, 2013 (90) | Y | Y | N | Y | Y | Y | Y | Y | Y | Good |
| Khosravi, 2013 (92) | Y | Y | Y | Y | Y | Y | Y | Y | Y | Good |
| Tosun, 2013 (248) | Y | N | Y | Y | Y | Y | Y | Y | Y | Good |
| Moosavy, 2013 (249) | Y | N | N | Y | Y | Y | Y | Y | Y | Fair |
| Kamkar, 2014 (250) | Y | Y | Y | Y | Y | Y | Y | Y | Y | Good |
| Rahimi, 2014 (251) | Y | N | Y | Y | Y | Y | Y | Y | Y | Good |
| Rahimirad, 2014 (252) | Y | N | Y | Y | Y | Y | Y | Y | Y | Good |
| Khodadadi, 2014 (253) | Y | Y | Y | Y | Y | Y | Y | Y | Y | Good |
| Kara, 2014 (254) | Y | Y | Y | Y | Y | Y | Y | N | Y | Good |
| Bakırdere, 2014 (115) | Y | N | Y | Y | Y | Y | Y | N | Y | Fair |
| Kocasari, 2014 (131) | Y | Y | N | Y | Y | Y | Y | Y | Y | Good |
| Temamogullari, 2014 (107) | Y | Y | Y | Y | Y | Y | Y | Y | Y | Good |
| Öztürk, 2014 (255) | Y | Y | Y | Y | Y | Y | Y | Y | Y | Good |
| Gul, 2014 (139) | Y | Y | Y | Y | Y | Y | Y | Y | Y | Good |
| Golge, 2014 (256) | Y | N | N | Y | Y | Y | Y | N | Y | Fair |
| Hassan, 2014 (157) | Y | Y | Y | Y | Y | Y | Y | Y | Y | Good |
| Christofidou, 2015 (257) | Y | N | Y | Y | Y | Y | Y | U | Y | Fair |
| Mwanza, 2015 (258) | Y | Y | Y | Y | Y | Y | Y | Y | Y | Good |
| El-kest, 2015 (259) | Y | Y | Y | Y | Y | Y | Y | Y | Y | Good |
| Elsayed, 2015 (260) | Y | N | N | Y | Y | Y | Y | U | Y | Fair |
| Mahmoudi, 2015 (37) | Y | N | Y | Y | Y | Y | Y | Y | Y | Good |
| Fallah, 2015 (261) | Y | Y | Y | Y | Y | Y | Y | Y | Y | Good |
| Barikbin, 2015 (262) | Y | Y | Y | Y | Y | Y | Y | Y | Y | Good |
| Zanjani, 2015 (263) | Y | Y | N | Y | Y | Y | Y | Y | Y | Good |
| Rouhi, 2015 (264) | Y | Y | Y | Y | Y | Y | Y | Y | Y | Good |
| Rezaei, 2015 (28) | Y | Y | Y | Y | Y | Y | Y | Y | Y | Good |
| Mason, 2015 (265) | Y | N | N | Y | Y | Y | Y | Y | Y | Fair |
| Mohajeri, 2015 (266) | Y | Y | N | Y | Y | Y | Y | Y | Y | Good |
| Kocak, 2015 (267) | Y | Y | N | Y | Y | Y | Y | N | Y | Fair |
| Sarica, 2015 (268) | Y | N | N | Y | Y | Y | Y | N | Y | Fair |
| Younis, 2016 (269) | Y | Y | Y | Y | N | Y | Y | Y | N | Fair |
| Hashemi, 2016 (270) | Y | U | Y | Y | N | Y | Y | Y | N | Fair |
| Dakhili, 2016 (271) | Y | Y | N | Y | Y | Y | Y | Y | Y | Good |
| Nikbakht, 2016 (272) | Y | Y | N | Y | Y | Y | Y | Y | Y | Good |
| Ghajarbeygi, 2016 (273) | Y | N | N | Y | Y | Y | Y | Y | Y | Fair |
| Bahrami, 2016 (65) | Y | Y | Y | Y | Y | Y | Y | Y | Y | Good |
| Sohrabi, 2016 (274) | Y | N | N | Y | Y | Y | Y | Y | Y | Fair |
| Mashak, 2016 (275) | Y | Y | N | Y | Y | Y | Y | Y | Y | Good |
| Fallah, 2016 (276) | Y | N | Y | Y | Y | Y | Y | Y | Y | Good |
| Taherabadi, 2016 (277) | Y | N | Y | Y | Y | Y | Y | N | Y | Fair |
| Mohammadi, 2016 (91) | Y | Y | N | Y | Y | Y | Y | Y | Y | Good |
| Tajik, 2016 (278) | Y | Y | Y | Y | Y | Y | Y | Y | Y | Good |
| Özgören, 2016 (279) | Y | Y | Y | Y | Y | Y | Y | Y | Y | Good |
| Sahin, 2016 (280) | Y | Y | Y | Y | Y | Y | Y | N | Y | Good |
| Omar, 2016 (281) | Y | N | Y | Y | Y | Y | Y | N | Y | Fair |
| Tahoun, 2017 (282) | Y | Y | N | Y | Y | Y | Y | Y | Y | Good |
| Koutamehr, 2017 (283) | Y | U | Y | Y | Y | Y | Y | Y | Y | Good |
| Shahbazi, 2017 (284) | Y | Y | Y | Y | Y | Y | Y | Y | Y | Good |
| Shokri, 2017 (285) | Y | N | N | N | Y | Y | Y | Y | Y | Fair |
| Sharifzadeh, 2017 (286) | Y | Y | Y | Y | Y | Y | Y | Y | Y | Good |
| Movassaghghazani, 2017 (287) | Y | Y | N | Y | Y | Y | Y | Y | Y | Good |
| Babolhavaegi, 2018 (288) | Y | Y | Y | Y | Y | Y | Y | N | Y | Good |
| Yildirm, 2018 (289) | Y | N | Y | Y | Y | Y | Y | Y | Y | Good |
| Öztürk Yilmazi, 2018 (290) | Y | N | N | Y | Y | Y | Y | N | Y | Fair |
| Madali, 2018 (291) | Y | Y | Y | Y | Y | Y | Y | Y | Y | Good |
| Sakin, 2018 (292) | Y | N | Y | Y | Y | Y | Y | Y | Y | Good |
| Hassan, 2018 (293) | Y | N | Y | Y | Y | Y | Y | Y | Y | Good |
| Shehab, 2019 (294) | Y | U | Y | Y | Y | Y | Y | Y | Y | Good |
| Zakaria, 2019 (73) | Y | Y | N | Y | Y | Y | Y | Y | Y | Good |
| Abdallah, 2019 (295) | Y | Y | N | Y | Y | Y | Y | N | Y | Good |
| Khaneghahi Abyaneh, 2019 (296) | Y | N | Y | Y | Y | Y | Y | Y | Y | Good |
| Abyaneh, 2019 (297) | Y | N | N | Y | Y | Y | Y | Y | Y | Fair |
| Mahmoodi Maymand, 2019 (298) | Y | Y | N | Y | Y | Y | Y | N | Y | Fair |
| Nejad, 2019 (299) | Y | N | N | Y | Y | Y | Y | Y | Y | Fair |
| Ansari, 2019 (300) | Y | N | Y | Y | Y | Y | Y | Y | Y | Good |
| Moghaddam, 2019 (101) | Y | Y | Y | Y | Y | Y | Y | N | Y | Good |
| Acaroz, 2019 (301) | Y | N | N | Y | Y | Y | Y | U | Y | Fair |
| Eker, 2019 (302) | Y | N | Y | Y | Y | Y | Y | N | Y | Fair |
| cetin, 2019 (303) | Y | N | N | Y | Y | Y | N | Y | Y | Poor |
| El-Tawab, 2020 (304) | Y | U | Y | Y | Y | Y | Y | N | Y | Fair |
| Ahmed, 2020 (305) | Y | Y | Y | Y | Y | Y | Y | Y | Y | Good |
| Ismaie, 2020 (306) | Y | Y | Y | Y | Y | Y | Y | Y | Y | Good |
| Hajmohammadi, 2020 (44) | Y | Y | N | Y | Y | Y | Y | Y | Y | Good |
| Ahmadi, 2020 (307) | Y | Y | N | Y | Y | Y | Y | Y | Y | Good |
| Khorshidi, 2020 (43) | Y | N | Y | Y | Y | Y | Y | Y | Y | Good |
| Mozaffari Nejad, 2020 (308) | Y | N | N | Y | Y | Y | Y | Y | Y | Fair |
| Heshmati, 2020 (309) | Y | Y | N | Y | Y | Y | Y | Y | Y | Good |
| Daou, 2020 (310) | Y | Y | Y | Y | Y | Y | Y | N | Y | Good |
| El Tawila, 2020 (311) | Y | Y | Y | Y | Y | Y | Y | Y | Y | Good |
| Murshed, 2020 (312) | Y | N | Y | N | Y | Y | Y | Y | Y | Fair |
| Adam, 2021 (313) | Y | Y | Y | Y | Y | Y | Y | Y | Y | Good |
| Jafari, 2021 (314) | Y | Y | Y | Y | Y | Y | Y | Y | Y | Good |
| Onmaz, 2021 (315) | Y | Y | Y | Y | Y | Y | Y | N | Y | Good |
| Esam, 2022, (316) | Y | N | Y | Y | Y | Y | Y | Y | Y | Good |
| Taşçi, 2022 (317) | Y | Y | N | Y | Y | Y | Y | Y | Y | Good |
| Mohamadin, 2022 (168) | Y | N | Y | Y | Y | Y | Y | Y | Y | Good |
| Khalifa, 2023 (318) | Y | N | N | Y | Y | Y | Y | Y | Y | Fair |
| Youssef, 2023 (319) | Y | U | N | Y | Y | Y | Y | Y | Y | Fair |
| Hamad, 2023 (320) | Y | Y | Y | Y | Y | Y | Y | Y | Y | Good |
| Footprint: N, no; U, unclear; Y, yes.  Q1. Appropriate sampling frame to address the target population, Q2. Appropriate sampling way of study participants, Q3. Adequate sample size, Q4. Detail description of study participants and settings, Q5. Data analysis with sufficient coverage of the identified sample, Q6. Use of valid methods to identify the condition, Q7. Standard, reliable way of measurement of condition for all participants, Q8. Availability of appropriate statistical analysis, Q9. Adequate response rate and management of low response rate | | | | | | | | | | |

# **Supplemental References:**

1. Ioannou‐Kakouri E, Christodoulidou M, Christou E, Constantinidou E. Immunoaffinity column/HPLC determination of aflatoxin M1 in milk. Food and Agricultural Immunology. 1995;7(2):131-7.

2. Hassanin NI. Detection of mycotoxigenic fungi and bacteria in processed cheese in Egypt. International biodeterioration & biodegradation. 1993;31(1):15-23.

3. Hamed AM, Abdel-Hamid M, Gámiz-Gracia L, García-Campaña AM, Arroyo-Manzanares N. Determination of aflatoxins in plant-based milk and dairy products by dispersive liquid–liquid microextraction and high-performance liquid chromatography with fluorescence detection. Analytical Letters. 2019;52(2):363-72.

4. Piekkola S, Turner P, Abdel-Hamid M, Ezzat S, El-Daly M, El-Kafrawy S, et al. Characterisation of aflatoxin and deoxynivalenol exposure among pregnant Egyptian women. Food Additives & Contaminants: Part A. 2012;29(6):962-71.

5. Badria FA. A multidisciplinary study to monitor mycotoxins in Egypt. Journal of Toxicology: Toxin Reviews. 1996;15(3):251-72.

6. Abdelhamid A. Occurrence of some mycotoxins (aflatoxin, ochratoxin A, citrinin, zearalenone and vomitoxin) in various Egyptian feeds. Archives of Animal Nutrition. 1990;40(7):647-64.

7. El-Sawi NM, El-Maghraby O, Mohran H, Abo-Gharbia M. Abnormal contamination of cottage cheese in Egypt. Journal of Applied Animal Research. 1994;6(1):81-90.

8. Megalla S, Hafez A. Detoxification of aflatoxin B1 by acidogenous yoghurt. Mycopathologia. 1982;77(2):89-91.

9. Hafez A, Megalla S, Mohran M, Nassar A. Aflatoxin and aflatoxicosis: V. The kinetic behaviour of dietary aflatoxins in colostrum drawn from cows postpartum. Mycopathologia. 1985;89(3):161-4.

10. El-Sayed AA, Soher EA, Neamat-Allah A. Human exposure to mycotoxins in Egypt. Mycotoxin research. 2002;18:23-30.

11. Kamel E, Bazalou M, Sdeek FA, Konuk M. Comparison of liquid chromatography instruments with single quadrupole and tandem mass spectrometry for trace level analysis: Aflatoxin m1 (afm1) in white cheese. International journal of food properties. 2017;20(sup2):2294-304.

12. Abdelmotilib N, Hamad G, Elderea H, Salem E, Sohaimy S. Aflatoxin M1 reduction in milk by a novel combination of probiotic bacterial and yeast strains. European Journal of Nutrition & Food Safety. 2018;8(2):83-99.

13. Wael F, El-Kady NN, Tayel AA. Infants exposure to aflatoxin M1 as a novel foodborne zoonosis. Food and Chemical Toxicology. 2011;49(11):2816-9.

14. Sebaei AS, Refai HM, Elbadry HT, Armeya SM. First risk assessment report of aflatoxins in Egyptian tahini. Journal of Food Composition and Analysis. 2020;92:103550.

15. Gouda G, Khattab H, Abdel-Wahhab M, El-Nor SA, El-Sayed H, Kholif S. Clay minerals as sorbents for mycotoxins in lactating goat’s diets: intake, digestibility, blood chemistry, ruminal fermentation, milk yield and composition, and milk aflatoxin M1 content. Small Ruminant Research. 2019;175:15-22.

16. Hassanin NI. Stability of aflatoxin M1 during manufacture and storage of yoghurt, yoghurt‐cheese and acidified milk. Journal of the Science of Food and Agriculture. 1994;65(1):31-4.

17. Farag MA, Jomaa SA, Abd El-Wahed A, R. El-Seedi H. The many faces of kefir fermented dairy products: Quality characteristics, flavour chemistry, nutritional value, health benefits, and safety. Nutrients. 2020;12(2):346.

18. El-Gohary I, Abd-Elsalam R, Fadal N, Mahmoud M. AFLATOXIN M1 INDUCING GENOTOXICITY AND PATHOLOGICAL LESIONS IN ORGAN MEATS, LIVER OF CATTLE SAMPLED FROM EL-BASATIN ABATTOIR, EGYPT. Journal of microbiology, biotechnology and food sciences. 2022;12(1):e3527-e.

19. Taherimaslak Z, Amoli-Diva M, Allahyary M, Pourghazi K. Magnetically assisted solid phase extraction using Fe3O4 nanoparticles combined with enhanced spectrofluorimetric detection for aflatoxin M1 determination in milk samples. Analytica Chimica Acta. 2014;842:63-9.

20. Jahangiri–Dehaghani F, Zare HR, Shekari Z. Measurement of aflatoxin M1 in powder and pasteurized milk samples by using a label–free electrochemical aptasensor based on platinum nanoparticles loaded on Fe–based metal–organic frameworks. Food chemistry. 2020;310:125820.

21. Abyaneh HK, Bahonar A, Noori N, Yazdanpanah H, AliAbadi MHS. The overall and variations of Aflatoxin M1 contamination of milk in Iran: A systematic review and meta-analysis study. Food chemistry. 2020;310:125848.

22. Jalili M, Scotter M. A review of aflatoxin M1 in liquid milk. Iranian Journal of Health, Safety and Environment. 2015;2(2):283-95.

23. Bodbodak S, Hesari J, Peighambardoust SH, Mahkam M. Selective decontamination of aflatoxin M1 in milk by molecularly imprinted polymer coated on the surface of stainless steel plate. International journal of dairy technology. 2018;71(4):868-78.

24. Hajmohammadi M, Valizadeh R, Naserian A, Nourozi ME, Oliveira CA. Effect of size fractionation of a raw bentonite on the excretion rate of aflatoxin M1 in milk from dairy cows fed with aflatoxin B1. International Journal of Dairy Technology. 2021;74(4):709-14.

25. Foroughi M, Sarabi Jamab M, Keramat J, Foroughi M. Immobilization of Saccharomyces cerevisiae on Perlite Beads for the Decontamination of Aflatoxin M1 in Milk. Journal of food science. 2018;83(7):2008-13.

26. Mahmoudi R, Zare P. Total and M1 aflatoxins contamination in meat and milk buffalo were slaughtered in the Northwest of Iran. 2014.

27. Kamkar A, Fallah AA, Mozaffari Nejad AS. The review of aflatoxin M1 contamination in milk and dairy products produced in Iran. Toxin Reviews. 2014;33(4):160-8.

28. Rezaei M, Fani A, Moini AL, Mirzajani P, Malekirad AA, Rafiei M, et al. Assessment of aflatoxin M1 levels in pasteurised milk, raw milk, and cheese in Arak, Iran. Toxin Reviews. 2015;34(2):61-5.

29. مجيد رپ, حميدرضا ت, مهدي را, حسن ر, محمدتقي ص. اندازه گيري ميزان آفلاتوكسين M1 در شيرهاي پاستوريزه.

30. رعیت ی, رامک, شکری, خسروی, ترابی. ارزیابی آلودگی میزان آفلاتوکسین M1 در نمونه های شیر خام به روش الایزا در استان یزد. مجله تحقیقات دامپزشکی (Journal of Veterinary Research). 2017;72(3):313-21.

31. Kamkar A. Detection of Aflatoxin M1 in UHT milk samples by ELISA. 2008.

32. Kamkar A, JAHED KGR, Alavi S. Occurrence of aflatoxin M1 in raw milk produced in Ardebil of Iran. 2011.

33. Pirestani A, Tabatabaei SN, Fazeli MH, Antikchi M, BAABAEI M. Comparison of HPLC and ELISA for determination of aflatoxin concentration in the milk and feeds of dairy cattle. 2011.

34. MOHAMMADIAN B, KHEZRI M, Ghasemipour N, Mafakheri S, POURGHAFOUR LP. Aflatoxin M1 contamination of raw and pasteurized milk produced in Sanandaj, Iran. 2010.

35. Rahimi E, Nilchian Z, Behzadnia A. Presence of aflatoxin M1 in pasteurized and UHT milk commercialized in Shiraz, Khuzestan and Yazd, Iran. 2011.

36. Jafarian-Dehkordi A, Pourradi N. Aflatoxin M1 contamination of human breast milk in Isfahan, Iran. Advanced Biomedical Research. 2013;2.

37. Mahmoudi R, Norian R. Aflatoxin B1 and M1 contamination in cow feeds and milk from Iran. Food and Agricultural Immunology. 2015;26(1):131-7.

38. Tabari M, Karim G, Ghavami M, Chamani M. Method validation for aflatoxin M1 determination in yoghurt using immunoaffinity column clean-up prior to high-performance liquid chromatography. Toxicology and Industrial Health. 2011;27(7):629-35.

39. Bayat M, Hashemi J, Razavilar V. Identification of dairy fungal contamination and reduction of aflatoxin M1 amount by three acid and bile resistant probiotic bacteria. Archives of Razi Institute. 2021;76(1):119.

40. Naeimipour F, Aghajani J, Kojuri SA, Ayoubi S. Useful approaches for reducing aflatoxin M1 content in milk and dairy products. Biomedical and biotechnology research journal (BBRJ). 2018;2(2):94-9.

41. Manoochehri M, Asgharinezhad AA, Safaei M. Determination of aflatoxin M1 in milk powder by ultrasonic-assisted extraction and dispersive solid-phase clean-up. Journal of chromatographic science. 2015;53(6):1000-6.

42. Hashemi M, Taherimaslak Z. Determination of aflatoxin M 1 in liquid milk using high performance liquid chromatography with fluorescence detection after magnetic solid phase extraction. Rsc Advances. 2014;4(63):33497-506.

43. Khorshidi M, Heshmati A, Hadian Z, Smaoui S, Mousavi Khaneghah A. The occurrence of aflatoxin M 1 in doogh, kefir, and kashk in Hamadan, Iran. Food Science and Technology. 2022;42.

44. Hajmohammadi M, Valizadeh R, Naserian A, Nourozi ME, Rocha RS, Oliveira CA. Composition and occurrence of aflatoxin M1 in cow's milk samples from Razavi Khorasan Province, Iran. International Journal of Dairy Technology. 2020;73(1):40-5.

45. Heshmati A, Ghadimi S, Ranjbar A, Khaneghah AM. Changes in aflatoxins content during processing of pekmez as a traditional product of grape. LWT. 2019;103:178-85.

46. Naghshbandi B, Omrani MA, Jafari-Sales A, Jalil AT, Naghsh N. Human Health Risk Assessment and Exposure Evaluation by Monte-Carlo Simulation Method for Aflatoxin M1 in Widely Consumed Infant Dried Powder Milk in Iran. Journal of Food Protection. 2023;86(3):100047-.

47. Mohammadi R, Erfani N, Sohrabvandi S, Mortazavi SA, Mortazavian AM, Sarlak Z, et al. Aflatoxin M1 reduction by probiotic strains in Iranian Feta cheese. Iranian Journal of Chemistry and Chemical Engineering. 2021;40(6):2069-78.

48. Hashemi SMB, Gholamhosseinpour A. Fermentation of table cream by Lactobacillus plantarum strains: Effect on fungal growth, aflatoxin M1 and ochratoxin A. International journal of food science & technology. 2019;54(2):347-53.

49. Khoshpey B, Farhud D, Zaini F. Aflatoxins in Iran: nature, hazards and carcinogenicity. Iranian journal of public health. 2011;40(4):1.

50. Sefidgar S, Mirzae M, Assmar M, Naddaf S. Aflatoxin M1 in pasteurized milk in Babol city, Mazandaran Province, Iran. Iranian journal of public health. 2011;40(1):115.

51. Ataee R, Tavana AM, Ataee M. Determination of Aflatoxin M1 Contamination and Integrity as well as Credibility. Iranian Journal of Public Health. 2012;41(10):97.

52. Ghiasian S, Maghsood A. Infants’ exposure to aflatoxin M1 from mother’s breast milk in Iran. Iranian journal of public health. 2012;41(3):119.

53. Mohammadi H, Alizadeh M, Bari M, Khosrowshahi A, Tajik H. Minimization of aflatoxin M1 content in Iranian white brine cheese. International journal of dairy technology. 2008;61(2):141-5.

54. MOHAMADI H, ALIZADEH M, RAHIMI J, QASRI S. RESEARCH Assessment of aflatoxin M1 levels in selected dairy products in north-western Iran. 2009.

55. Abnous K, Danesh NM, Ramezani M, Alibolandi M, Nameghi MA, Zavvar TS, et al. A novel colorimetric aptasensor for ultrasensitive detection of aflatoxin M1 based on the combination of CRISPR-Cas12a, rolling circle amplification and catalytic activity of gold nanoparticles. Analytica Chimica Acta. 2021;1165:338549.

56. Kamkar A, Karim G, Aliabadi FS, Khaksar R. Fate of aflatoxin M1 in Iranian white cheese processing. Food and Chemical Toxicology. 2008;46(6):2236-8.

57. Fallah AA, Jafari T, Fallah A, Rahnama M. Determination of aflatoxin M1 levels in Iranian white and cream cheese. Food and chemical toxicology. 2009;47(8):1872-5.

58. Gandomi H, Misaghi A, Basti AA, Bokaei S, Khosravi A, Abbasifar A, et al. Effect of Zataria multiflora Boiss. essential oil on growth and aflatoxin formation by Aspergillus flavus in culture media and cheese. Food and chemical toxicology. 2009;47(10):2397-400.

59. Hooshfar S, Khosrokhavar R, Yazdanpanah H, Eslamizad S, Kobarfard F, Nazari F, et al. Health risk assessment of aflatoxin M1 in infant formula milk in IR Iran. Food and Chemical Toxicology. 2020;142:111455.

60. Sadeghi N, Oveisi MR, Jannat B, Hajimahmoodi M, Bonyani H, Jannat F. Incidence of aflatoxin M1 in human breast milk in Tehran, Iran. Food control. 2009;20(1):75-8.

61. Heshmati A, Milani JM. Contamination of UHT milk by aflatoxin M1 in Iran. Food Control. 2010;21(1):19-22.

62. Fallah AA. Aflatoxin M1 contamination in dairy products marketed in Iran during winter and summer. Food control. 2010;21(11):1478-81.

63. Fallah AA, Rahnama M, Jafari T, Saei-Dehkordi SS. Seasonal variation of aflatoxin M1 contamination in industrial and traditional Iranian dairy products. Food Control. 2011;22(10):1653-6.

64. Afshar P, Shokrzadeh M, Kalhori S, Babaee Z, Saravi SS. Occurrence of Ochratoxin A and Aflatoxin M1 in human breast milk in Sari, Iran. Food Control. 2013;31(2):525-9.

65. Bahrami R, Shahbazi Y, Nikousefat Z. Aflatoxin M1 in milk and traditional dairy products from west part of Iran: occurrence and seasonal variation with an emphasis on risk assessment of human exposure. Food Control. 2016;62:250-6.

66. Sarlak Z, Rouhi M, Mohammadi R, Khaksar R, Mortazavian AM, Sohrabvandi S, et al. Probiotic biological strategies to decontaminate aflatoxin M1 in a traditional Iranian fermented milk drink (Doogh). Food control. 2017;71:152-9.

67. Sarmast E, Fallah AA, Jafari T, Khaneghah AM. Impacts of unit operation of cheese manufacturing on the aflatoxin M1 level: a global systematic review and meta-analysis. LWT. 2021;148:111772.

68. Hashemi M, Taherimaslak Z, Rashidi S. Enhanced spectrofluorimetric determination of aflatoxin M1 in liquid milk after magnetic solid phase extraction. Spectrochimica Acta Part A: Molecular and Biomolecular Spectroscopy. 2014;128:583-90.

69. Amoli-Diva M, Taherimaslak Z, Allahyari M, Pourghazi K, Manafi MH. Application of dispersive liquid–liquid microextraction coupled with vortex-assisted hydrophobic magnetic nanoparticles based solid-phase extraction for determination of aflatoxin M1 in milk samples by sensitive micelle enhanced spectrofluorimetry. Talanta. 2015;134:98-104.

70. Fakhri Y, Rahmani J, Oliveira CAF, Franco LT, Corassin CH, Saba S, et al. Aflatoxin M1 in human breast milk: A global systematic review, meta-analysis, and risk assessment study (Monte Carlo simulation). Trends in food science & technology. 2019;88:333-42.

71. Danesh NM, Bostan HB, Abnous K, Ramezani M, Youssefi K, Taghdisi SM, et al. Ultrasensitive detection of aflatoxin B1 and its major metabolite aflatoxin M1 using aptasensors: A review. TrAC Trends in Analytical Chemistry. 2018;99:117-28.

72. Karimi Dehcheshmeh B, Shakerian A, Rahimi E. Evaluation of aflatoxin M1 and heavy metal in raw materials and infant formula produced in Pegah dairy plants, Iran. Journal of Chemical Health Risks. 2021;11(1):55-62.

73. Zakaria AM, Amin YA, Khalil OSF, Abdelhiee EY, Elkamshishi MM. Rapid detection of aflatoxin M1 residues in market milk in Aswan Province, Egypt and effect of probiotics on its residues concentration. Journal of advanced veterinary and animal research. 2019;6(2):197.

74. Karimi DB, SHAKERIAN A, RAHIMI E. Investigation of Heavy Metals, Aflatoxin M1 and Physicochemical Properties of Milk Used in Baby Dry Milk Formula in Shahrekord City. 2022.

75. محمدحسين م, سعيد آو. مطالعه ميزان آفلاتوکسين M1 در شير خام مراکز جمع آوري شير در شهر تبريز.

76. Khoori E, Hakimzadeh V, Mohammadi Sani A, Rashidi H. Effect of ozonation, UV light radiation, and pulsed electric field processes on the reduction of total aflatoxin and aflatoxin M1 in acidophilus milk. Journal of food processing and preservation. 2020;44(10):e14729.

77. Gholipour M, Alinia F, Babaee Z. Determination of aflatoxin m1 in milk processed in mazandaran dairy factorie. J Mazandaran Univ Med Sci. 2012;22(93):39-46.

78. Moeinian K, Rastgoo T. Seasonal variation of aflatoxin M1contamination in raw, pasteurized and school milk in Shahrood, Iran. Journal of Mazandaran University of Medical Sciences. 2014;24(119):19-28.

79. Ghariby H, Takdastan A, Neisi AK, Rezazadeh H, Kuhpaee H. Investigating aflatoxin M1 contamination in buffalos milk using immunoassay. Journal of Mazandaran University of Medical Sciences. 2017;26(145):248-56.

80. نجفیان, محمود, نجفیان. بررسی میزان افلاتوکسین M1 در نمونه های شیر کارخانه های لبنیاتی گیلان به روش الایزا. فصلنامه علمی پژوهشی دنیای میکروب ها. 2015;8(شماره 3 (پیاپی 24)):248-53.

81. Gholamalian R, Mahdavi AH, Riasi A. Hepatic fatty acids profile, oxidative stability and egg quality traits ameliorated by supplementation of alternative lipid sources and milk thistle meal. Journal of Animal Physiology and Animal Nutrition. 2022;106(4):860-71.

82. Amiridumari H, Sarir H, Afzali N, FaniMakki O. Effects of milk thistle seed against aflatoxin B1 in broiler model. Journal of research in medical sciences: the official journal of Isfahan University of Medical Sciences. 2013;18(9):786.

83. Kamkar A. A study on the occurrence of aflatoxin M1 in Iranian Feta cheese. Food control. 2006;17(10):768-75.

84. اله مخ, كاميار ي, راهب ق. غلظت آفلاتوكسين M1 در شير خام توليدي در شهرستان هاي استان سمنان.

85. احمدی, الاسلامی ح, محمد, کیانی, مولوی, هومان, et al. شناسایی آفلاتوکسین M1 در شیر به کمک آپتاسنسور الکتروشیمیایی مبتنی بر الکترود صفحه-چاپی و روش ولتامتری چرخه‌ای. مجله علوم و صنایع غذایی ایران. 2021;18(114):15-23.

86. Bahrami R, Shahbazi Y, Nikousefat Z. Occurrence and seasonal variation of aflatoxin in dairy cow feed with estimation of aflatoxin M 1 in milk from Iran. Food and Agricultural Immunology. 2016;27(3):388-400.

87. Oveisi M-R, Jannat B, Sadeghi N, Hajimahmoodi M, Nikzad A. Presence of aflatoxin M1 in milk and infant milk products in Tehran, Iran. Food Control. 2007;18(10):1216-8.

88. Sheini A. Colorimetric aggregation assay based on array of gold and silver nanoparticles for simultaneous analysis of aflatoxins, ochratoxin and zearalenone by using chemometric analysis and paper based analytical devices. Microchimica Acta. 2020;187:1-11.

89. Mahdavi R, Nikniaz L, Arefhosseini S, Vahed Jabbari M. Determination of aflatoxin M 1 in breast milk samples in Tabriz–Iran. Maternal and Child Health Journal. 2010;14:141-5.

90. Riahi-Zanjani B, Balali-Mood M. Aflatoxin M 1 contamination in commercial pasteurized milk from local markets in Fariman, Iran. Mycotoxin research. 2013;29:271-4.

91. Mohammadi H, Shokrzadeh M, Aliabadi Z, Riahi-Zanjani B. Occurrence of aflatoxin M 1 in commercial pasteurized milk samples in Sari, Mazandaran province, Iran. Mycotoxin research. 2016;32:85-7.

92. Khosravi AR, Shokri H, Eshghi S, Darvishi S. Global occurrence of aflatoxin M 1 in milk with particular reference to Iran. Food security. 2013;5:533-9.

93. Riahi-Zanjani B, Heidarzadegan M, Badibostan H, Karimi G. Determination of 17β-estradiol in commercial pasteurized and sterilized milk samples in Mashhad, Iran. Journal of food science and technology. 2019;56:4795-8.

94. Hassanpour M, Rezaie MR, Baghizadeh A. Practical analysis of aflatoxin M1 reduction in pasteurized Milk using low dose gamma irradiation. Journal of Environmental Health Science and Engineering. 2019;17:863-72.

95. Pardakhti A, Maleki S. Risk assessment of Aflatoxin M1 contamination of milk in Iran. International Journal of Environmental Research. 2019;13:265-71.

96. Beitollahi H, Tajik S, Dourandish Z, Zhang K, Le QV, Jang HW, et al. Recent advances in the aptamer-based electrochemical biosensors for detecting aflatoxin B1 and its pertinent metabolite aflatoxin M1. Sensors. 2020;20(11):3256.

97. Mason S, Arjmandtalab S, Khosravi Arsanjani A, Hajimohammadi B, Rahimzade A, Ehrampoush M, et al. Reduction of Aflatoxin M 1 during Production of Kashk, a Traditional Iranian Dairy Product. Journal of Food Quality & Hazards Control. 2016;3(1).

98. Tajkarimi M, Aliabadi FS, Nejad MS, Pursoltani H, Motallebi A, Mahdavi H. Seasonal study of aflatoxin M1 contamination in milk in five regions in Iran. International journal of food microbiology. 2007;116(3):346-9.

99. Tajkarimi M, Aliabadi-Sh F, Nejad AS, Poursoltani H, Motallebi A, Mahdavi H. Aflatoxin M1 contamination in winter and summer milk in 14 states in Iran. Food Control. 2008;19(11):1033-6.

100. عزیزی عغ, خوشنویس سح, هاشمی سج. سنجش آفلاتوکسین M1 در شیر پاستوریزه و استریلیزه مصرفی شهر بابل. مجله دانشکده پزشکی دانشگاه علوم پزشکی تهران. 2008;65(13):20-4.

101. Moghaddam AF, Rychlik M, Hosseini H, Janat B, Yazdanpanah H, AliAbadi MS. Risk associated with the intake of aflatoxin M1 from milk in Iran. World Mycotoxin Journal. 2019;12(2):191-200.

102. Pour SH, Mahmoudi S, Masoumi S, Rezaie S, Barac A, Ranjbaran M, et al. Aflatoxin M1 contamination level in Iranian milk and dairy products: A systematic review and meta-analysis. World mycotoxin journal. 2020;13(1):67-82.

103. Al-Sawaf S, Abdullah O, Sheet O. Use of Enzyme Linked Immunosorbent Assay for detection of aflatoxin M1 in milk powder. Iraqi Journal of Veterinary Sciences. 2012;26(1):39-42.

104. Mayer A, Poljakoff-mayber A, Robinson P, Slowatizky I. Simple bioassay for detection of aflatoxin in milk. Toxicon. 1969;7(1):13-4.

105. Britzi M, Friedman S, Miron J, Solomon R, Cuneah O, Shimshoni JA, et al. Carry-over of aflatoxin B1 to aflatoxin M1 in high yielding Israeli cows in mid-and late-lactation. Toxins. 2013;5(1):173-83.

106. Deveci O, Sezgin E. Changes in concentration of aflatoxin M1 during manufacture and storage of skim milk powder. Journal of food protection. 2006;69(3):682-5.

107. Temamogullari F, Kanici A. Aflatoxin M1 in dairy products sold in Şanlıurfa, Turkey. Journal of Dairy Science. 2014;97(1):162-5.

108. Er B, Demirhan B, Yentür G. Investigation of aflatoxin M1 levels in infant follow-on milks and infant formulas sold in the markets of Ankara, Turkey. Journal of Dairy Science. 2014;97(6):3328-31.

109. Elmali M, Yapar K, Kart A, Yaman H. Aflatoxin M1 levels in milk powder consumed in Turkey. Journal of Animal and Veterinary Advances. 2008;7(5):643-5.

110. Oruc HH, Cibik R, Yilmaz E, Kalkanli O. Distribution and stability of aflatoxin M1 during processing and ripening of traditional white pickled cheese. Food Additives and Contaminants. 2006;23(2):190-5.

111. Kabak B, Var I. Factors affecting the removal of aflatoxin M1 from food model by Lactobacillus and Bifidobacterium strains. Journal of environmental science and health, part B. 2008;43(7):617-24.

112. Gürbay A, Engin AB, Çağlayan A, Şahin G. Aflatoxin M1 levels in commonly consumed cheese and yogurt samples in Ankara, Turkey. Ecology of Food and Nutrition. 2006;45(6):449-59.

113. Sarimehmetoğlu B, Küplülü Ö. Binding ability of aflatoxin M1 to yoghurt bacteria. Ankara Üniversitesi Veteriner Fakültesi Dergisi. 2004;51(3):195-8.

114. Baydar T, Erkekoglu P, Sipahi H, Sahin G. Aflatoxin B1, M1 and ochratoxin A levels in infant formulae and baby foods marketed in Ankara, Turkey. Journal of Food and Drug Analysis. 2007;15(1):11.

115. Bakırdere S, Yaroğlu T, Tırık N, Demiröz M, Karaca A. Determination of trace aflatoxin M1 levels in milk and milk products consumed in Turkey by using enzyme-linked immunosorbent assay. Food and Agricultural Immunology. 2014;25(1):61-9.

116. Aycicek H, Aksoy A, Saygi S. Determination of aflatoxin levels in some dairy and food products which consumed in Ankara, Turkey. Food Control. 2005;16(3):263-6.

117. Kivanç M. Fungal contamination of Kashar cheese in Turkey. Food/Nahrung. 1992;36(6):578-83.

118. Er Demirhan B, Demirhan B. The investigation of mycotoxins and Enterobacteriaceae of cereal-based baby foods marketed in Turkey. Foods. 2021;10(12):3040.

119. Tasci F. Microbiological and chemical properties of raw milk consumed in Burdur. J Anim Vet Adv. 2011;10(5):635-41.

120. Guzel‐Seydim Z, Seydim A, Greene A, Taş T. Determination of antimutagenic properties of acetone extracted fermented milks and changes in their total fatty acid profiles including conjugated linoleic acids. International journal of dairy technology. 2006;59(3):209-15.

121. Var I, Kabak B. Detection of aflatoxin M1 in milk and dairy products consumed in Adana, Turkey. International Journal of Dairy Technology. 2009;62(1):15-8.

122. Gürbay A, Sabuncuoğlu SA, Girgin G, Şahin G, Yiğit Ş, Yurdakök M, et al. Exposure of newborns to aflatoxin M1 and B1 from mothers’ breast milk in Ankara, Turkey. Food and chemical toxicology. 2010;48(1):314-9.

123. Yaroglu T, Oruc H, Tayar M. Aflatoxin M1 levels in cheese samples from some provinces of Turkey. Food Control. 2005;16(10):883-5.

124. Tekinşen KK, Uçar G. Aflatoxin M1 levels in butter and cream cheese consumed in Turkey. Food Control. 2008;19(1):27-30.

125. Ardic M, Karakaya Y, Atasever M, Adiguzel G. Aflatoxin M1 levels of Turkish white brined cheese. Food Control. 2009;20(3):196-9.

126. Kabak B. Aflatoxin M1 and ochratoxin A in baby formulae in Turkey: Occurrence and safety evaluation. Food Control. 2012;26(1):182-7.

127. Colak H, Hampikyan H, Bingol EB. Some residues and contaminants in milk and dairy products. Asian Journal of Chemistry. 2007;19(3):1789.

128. Virdis S, Corgiolu G, Scarano C, Pilo AL, De Santis EPL. Occurrence of aflatoxin M1 in tank bulk goat milk and ripened goat cheese. Food Control. 2008;19(1):44-9.

129. Atasever MA, Atasever M, ÖZTURAN K. Aflatoxin M1 levels in retail yoghurt and ayran in Erzurum in Turkey. Turkish Journal of Veterinary & Animal Sciences. 2011;35(1):59-62.

130. Kılıç Altun S, Gürbüz S, Ayağ E. Aflatoxin M 1 in human breast milk in southeastern Turkey. Mycotoxin Research. 2017;33:103-7.

131. Sahindokuyucu Kocasari F. Occurrence of aflatoxin M 1 in UHT milk and infant formula samples consumed in Burdur, Turkey. Environmental monitoring and assessment. 2014;186:6363-8.

132. Nilüfer D, Boyacıoǧlu D. Comparative study of three different methods for the determination of aflatoxins in tahini. Journal of Agricultural and Food Chemistry. 2002;50(12):3375-9.

133. Aygun O, Essiz D, Durmaz H, Yarsan E, Altintas L. Aflatoxin M 1 levels in Surk samples, a traditional Turkish cheese from southern Turkey. Bulletin of environmental contamination and toxicology. 2009;83:164-7.

134. Torlak E, Akan I. Aflatoxin contamination in tahini. Quality Assurance and Safety of Crops & Foods. 2013;5(3):221-5.

135. ATASEVER MA, Özlü H, Istanbullugil FR, Atasever M. Determination of AFM1 levels of Mare’s milk and koumiss produced in the highlands of the Kyrgyz Republic. Kafkas Üniversitesi Veteriner Fakültesi Dergisi. 2021;27(1):37-42.

136. Aksoy A, Atmaca E, Yazici F, GÜVENÇ D, GÜL O, DERVİŞOĞLU M. Comparative analysis of aflatoxin M1 in marketed butter by ELISA and HPLC. Kafkas Üniversitesi Veteriner Fakültesi Dergisi. 2016;22(4).

137. Sanli T, Deveci O, Sezgin E. Effects of pasteurization and storage on stability of aflatoxin M1 in yogurt. Kafkas Üniversitesi Veteriner Fakültesi Dergisi. 2012;18(6).

138. ATASEVER M, OZTURAN K, URCAR S. Determination of aflatoxin M1 level in butter samples consumed in Erzurum, Turkey. Kafkas Üniversitesi Veteriner Fakültesi Dergisi. 2010;16(1).

139. Gul O, Dervisoglu M. Occurrence of aflatoxin M1 in vacuum packed kashar cheeses in Turkey. International Journal of Food Properties. 2014;17(2):273-82.

140. Dinckaya E, Kınık Ö, Sezgintürk MK, Altuğ Ç, Akkoca A. Development of an impedimetric aflatoxin M1 biosensor based on a DNA probe and gold nanoparticles. Biosensors and Bioelectronics. 2011;26(9):3806-11.

141. Kabak B. Aflatoxins in foodstuffs: Occurrence and risk assessment in Turkey. Journal of Food Composition and Analysis. 2021;96:103734.

142. Atasever M, Yildirim Y, Atasever M, Tastekin A. Assessment of aflatoxin M1 in maternal breast milk in Eastern Turkey. Food and Chemical Toxicology. 2014;66:147-9.

143. KIVANC M. Mold growth and presence of aflatoxin in some Turkish cheeses. Journal of Food Safety. 1990;10(4):287-94.

144. Sevim S, Topal GG, Tengilimoglu-Metin MM, Sancak B, Kizil M. Effects of inulin and lactic acid bacteria strains on aflatoxin M1 detoxification in yoghurt. Food control. 2019;100:235-9.

145. Ayar A, SERT D, Con AH. A study on the occurrence of aflatoxin in raw milk due to feeds. Journal of food safety. 2007;27(2):199-207.

146. Akgönüllü S, Yavuz H, Denizli A. Development of gold nanoparticles decorated molecularly imprinted–based plasmonic sensor for the detection of aflatoxin M1 in milk samples. Chemosensors. 2021;9(12):363.

147. Kav K, Col R, Tekinsen KK. Detection of aflatoxin M1 levels by ELISA in white-brined Urfa cheese consumed in Turkey. Food control. 2011;22(12):1883-6.

148. Colak H. Determination of aflatoxin M1 levels in Turkish White and Kashar cheeses made of experimentally contaminated raw milk. Journal of Food and Drug Analysis. 2007;15(2):9.

149. Oruc HH, Cibik R, Yilmaz E, Gunes E. Fate of aflatoxin M1 in Kashar cheese. Journal of food safety. 2007;27(1):82-90.

150. Deveci O. Changes in the concentration of aflatoxin M1 during manufacture and storage of White Pickled cheese. Food Control. 2007;18(9):1103-7.

151. Awaisheh SS, Rahahleh RJ, Algroom RM, Ala’a A, Ja’far M, Al-Dababseh BA. Contamination level and exposure assessment to Aflatoxin M1 in Jordanian infant milk formulas. Italian journal of food safety. 2019;8(3).

152. Omar SS. Incidence of aflatoxin M1 in human and animal milk in Jordan. Journal of Toxicology and Environmental Health, Part A. 2012;75(22-23):1404-9.

153. Bani Ismail Z, Al-Nabulsi F, Abu-Basha E, Hananeh W. Occurrence of on-farm risk factors and health effects of mycotoxins in dairy farms in Jordan. Tropical Animal Health and Production. 2020;52:2371-7.

154. Dashti B, Al-Hamli S, Alomirah H, Al-Zenki S, Abbas AB, Sawaya W. Levels of aflatoxin M1 in milk, cheese consumed in Kuwait and occurrence of total aflatoxin in local and imported animal feed. Food Control. 2009;20(7):686-90.

155. Assaf JC, Khoury AE, Chokr A, Louka N, Atoui A. A novel method for elimination of aflatoxin M1 in milk using Lactobacillus rhamnosus GG biofilm. International journal of dairy technology. 2019;72(2):248-56.

156. Elkak A, El Atat O, Habib J, Abbas M. Occurrence of aflatoxin M1 in cheese processed and marketed in Lebanon. Food Control. 2012;25(1):140-3.

157. Hassan HF, Kassaify Z. The risks associated with aflatoxins M1 occurrence in Lebanese dairy products. Food Control. 2014;37:68-72.

158. Elaridi J, Dimassi H, Hassan H. Aflatoxin M1 and ochratoxin A in baby formulae marketed in Lebanon: Occurrence and safety evaluation. Food Control. 2019;106:106680.

159. Daou R, Hoteit M, Bookari K, Al-Khalaf M, Nahle S, Al-Jawaldeh A, et al. Aflatoxin B1 occurrence in children under the age of five’s food products and aflatoxin M1 exposure assessment and risk characterization of Arab infants through consumption of infant powdered formula: a Lebanese experience. Toxins. 2022;14(5):290.

160. Al Zuheir IM, Omar JA. Presence of aflatoxin M1 in raw milk for human consumption in Palestinian. Walailak Journal of Science and Technology (WJST). 2012;9(3):201-5.

161. Al Jabir M, Barcaru A, Latiff A, Jaganjac M, Ramadan G, Horvatovich P. Dietary exposure of the Qatari population to food mycotoxins and reflections on the regulation limits. Toxicology Reports. 2019;6:975-82.

162. Ul Hassan Z, Al Thani R, A. Atia F, Al Meer S, Migheli Q, Jaoua S. Co-occurrence of mycotoxins in commercial formula milk and cereal-based baby food on the Qatar market. Food Additives & Contaminants: Part B. 2018;11(3):191-7.

163. Chrouda A, Ayed D, Zinoubi K, Majdoub H, Jaffrezic-Renault N. Highly stable and ultra-sensitive amperometric aptasensor based on pectin stabilized gold nanoparticles on graphene oxide modified GCE for the detection of aflatoxin M1. Food Chemistry Advances. 2022;1:100068.

164. Aly M, Al-Seeni M, Qustib S, El-Sawi N. Mineral content and microbiological examination of some white cheese in Jeddah. Saudi Arabia during summer. 2008:3031-4.

165. Elsanhoty RM, Salam SA, Ramadan MF, Badr FH. Detoxification of aflatoxin M1 in yoghurt using probiotics and lactic acid bacteria. Food control. 2014;43:129-34.

166. Hashem A, Abd-Allah EF. Natural Occurrence of Aflatoxin M-1 in Egyptian Milk and Cheese. JOURNAL OF PURE AND APPLIED MICROBIOLOGY. 2013;7(3):1769-73.

167. Abdulrazzaq YM, Osman N, Yousif ZM, Al-Falahi S. Aflatoxin M1 in breast-milk of UAE women. Annals of tropical paediatrics. 2003;23(3):173-9.

168. Mohamadin M, Rama A, Seboussi R. Aflatoxin M1 in Ultra High Temperature Milk Consumed in Sharjah, United Arab Emirates. Journal of food quality and hazards control. 2022.

169. AL‐ZENKI S, AL‐MAZEEDI H, AL‐HOOTI S, AL‐ATTI T, AL‐MUTAWAH Q, Alomirah H, et al. Quality and safety characteristics of milk sold in the state of Kuwait. Journal of food processing and preservation. 2007;31(6):702-13.

170. Saad A, Abdelgadir A, Moss M. Aflatoxin in human and camel milk in Abu Dhabi, United Arab Emirates. Mycotoxin Research. 1989;5(2):57-60.

171. Haydar M, Benelli L, Brera C. Occurrence of aflatoxins in Syrian foods and foodstuffs: a preliminary study. Food chemistry. 1990;37(4):261-8.

172. Ioannou-Kakouri E, Aletrari M, Christou E, Hadjioannou-Ralli A, Koliou A, Akkelidou D. Surveillance and control of aflatoxins B1, B2, G1, G2, and M1 in foodstuffs in the Republic of Cyprus: 1992–1996. Journal of AOAC International. 1999;82(4):883-92.

173. Bakirci I. A study on the occurrence of aflatoxin M1 in milk and milk products produced in Van province of Turkey. Food control. 2001;12(1):47-51.

174. Ivastava Sr V, Bu-Abbas A, Al-Johar W, Al-Mufti S, Siddiqui M. Aflatoxin M 1 contamination in commercial samples of milk and dairy products in Kuwait. Food Additives & Contaminants. 2001;18(11):993-7.

175. Hismiogullari S, Basalan M, Hismiogullari A. Prevalence of mold growth and aflatoxin M1 in Kashar and white cheese produced in Western Turkey. Milchwissenschaft. 2003;58(7-8).

176. Gürses M, ERDOĞAN A, Çetin B. Occurrence of aflatoxin M_1 in some cheese types sold in erzurum, Turkey. Turkish Journal of Veterinary & Animal Sciences. 2004;28(3):527-30.

177. Sarımehmetoglu B, Kuplulu O, Celik TH. Detection of aflatoxin M1 in cheese samples by ELISA. Food control. 2004;15(1):45-9.

178. Kamkar A. A study on the occurrence of aflatoxin M1 in raw milk produced in Sarab city of Iran. Food control. 2005;16(7):593-9.

179. Çelik TH, Sarımehmetoğlu B, Küplülü Ö. Aflatoxin M1 contamination in pasteurised milk. Veterinarski arhiv. 2005;75(1):57-65.

180. Kamber U. AFLATOXIN M~ 1 CONTAMINATION OF SOME COMMERCIAL TURKISH CHEESES FROM MARKETS IN KARS, TURKEY. Fresenius Environmental Bulletin. 2005;14(11):1046.

181. Tekinşen KK, Tekinşen OC. Aflatoxin M1 in white pickle and Van otlu (herb) cheeses consumed in southeastern Turkey. Food Control. 2005;16(7):565-8.

182. Alborzi S, Rashidi M, Astaneh B. Aflatoxin M1 contamination in pasteurized milk in Shiraz (south of Iran). Food Control. 2006;17(7):582-4.

183. Akkaya L, Birdane YO, Oguz H, Cemek M. Occurrence of aflatoxin M~ 1 in yogurt samples from Afyonkarahisar, Turkey. Bulletin-Veterinary Institute in Pulawy. 2006;50(4):517.

184. Bașkaya R, Aydın A, Yıldız A, Bostan K. Aflatoxin M1 levels of some cheese varieties in Turkey. Medycyna Weterynaryjna. 2006;62(7):778-80.

185. Unusan N. Occurrence of aflatoxin M1 in UHT milk in Turkey. Food and Chemical Toxicology. 2006;44(11):1897-900.

186. Gürbay A, Aydın S, Girgin G, Engin A, Şahin G. Assessment of aflatoxin M1 levels in milk in Ankara, Turkey. Food control. 2006;17(1):1-4.

187. Colak H, Hampikyan H, Ulusoy B, Ergun O. Comparison of a competitive ELISA with an HPLC method for the determination of aflatoxin M1 in Turkish White, Kasar and Tulum cheeses. European Food Research and Technology. 2006;223:719-23.

188. Tajik H, Rohani SMR, Moradi M. Milk in Urmia, Iran. Pakistan Journal of Biological Sciences. 2007;10(22):4103-7.

189. Karimi G, Hassanzadeh M, Teimuri M, Nazari F, Nili A. Aflatoxin M1 contamination in pasteurized milk in Mashhad, Iran. Iranian Journal of Pharmaceutical Sciences. 2007;3(3):153-6.

190. Ghiasian SA, Maghsood AH, Neyestani TR, Mirhendi SH. Occurrence of aflatoxin M1 in raw milk during the summer and winter seasons in Hamedan, Iran. Journal of Food Safety. 2007;27(2):188-98.

191. Oezdemir M. Determination of aflatoxin M1 levels in goat milk consumed in Kilis province. Ankara Üniversitesi Veteriner Fakültesi Dergisi. 2007;54(2):99-103.

192. Yapar K, Elmali M, Kart A, Yaman H. Aflatoxin M1 levels in different type of cheese products produced in Turkey. Medycyna Wet. 2008;64(1):53-5.

193. Tekinşen KK, Eken HS. Aflatoxin M1 levels in UHT milk and kashar cheese consumed in Turkey. Food and Chemical Toxicology. 2008;46(10):3287-9.

194. Akkaya L, Birdane Y, Cemek M, Oguz H. OCCURANCE OF AFLATOXIN M. The Indian Veterinary Journal. 2009;86:107-8.

195. Motawee MM, Bauer J, McMahon DJ. Survey of aflatoxin M 1 in cow, goat, buffalo and camel milks in Ismailia-Egypt. Bulletin of Environmental Contamination and Toxicology. 2009;83:766-9.

196. Ghazani MHM. Aflatoxin M1 contamination in pasteurized milk in Tabriz (northwest of Iran). Food and Chemical Toxicology. 2009;47(7):1624-5.

197. Movassagh MH. Presence of Aflatoxin M1 Ewe’s Milk in the Northwest Region of Iran. Journal of Applied Biological Sciences. 2009;3(3):17-9.

198. Rahimi E, Shakerian A, Jafariyan M, Ebrahimi M, Riahi M. Occurrence of aflatoxin M1 in raw, pasteurized and UHT milk commercialized in Esfahan and Shahr-e Kord, Iran. Food Security. 2009;1(3):317-20.

199. Rahimi E, Karim G, Shakerian A. Occurrence of aflatoxin M₁ in traditional cheese consumed in Esfahan, Iran. 2009.

200. Ardic M. Occurrence of aflatoxin M1 in raw ewe's milk produced in Sanliurfa, Turkey. Asian Journal of Chemistry. 2009;21(3):1966.

201. Gündinç U, Filazi A. Detection of aflatoxin M1 concentrations in UHT milk consumed in Turkey markets by ELISA. Pakistan journal of biological sciences: PJBS. 2009;12(8):653-6.

202. Arslan C, EŞSİZ D. Nutrient composition and mycotoxin residues in the hay stored as stack forms during the storage period, and aflatoxin M1 in the milk of the cows fed by them. Kafkas Üniversitesi Veteriner Fakültesi Dergisi. 2009;15(5).

203. Herzallah SM. Determination of aflatoxins in eggs, milk, meat and meat products using HPLC fluorescent and UV detectors. Food Chemistry. 2009;114(3):1141-6.

204. Ghanem I, Orfi M. Aflatoxin M1 in raw, pasteurized and powdered milk available in the Syrian market. Food Control. 2009;20(6):603-5.

205. Fallah AA. Assessment of aflatoxin M1 contamination in pasteurized and UHT milk marketed in central part of Iran. Food and Chemical Toxicology. 2010;48(3):988-91.

206. Mohamadi H, Alizadeh M, Rahimi J, Qasri S. Assessment of aflatoxin M1 levels in selected dairy products in north‐western Iran. International journal of dairy technology. 2010;63(2):262-5.

207. Rahimi E, Bonyadian M, Rafei M, Kazemeini H. Occurrence of aflatoxin M1 in raw milk of five dairy species in Ahvaz, Iran. Food and Chemical Toxicology. 2010;48(1):129-31.

208. Sani AM, Nikpooyan H, Moshiri R. Aflatoxin M1 contamination and antibiotic residue in milk in Khorasan province, Iran. Food and Chemical Toxicology. 2010;48(8-9):2130-2.

209. Nemati M, Mehran MA, Hamed PK, Masoud A. A survey on the occurrence of aflatoxin M1 in milk samples in Ardabil, Iran. Food control. 2010;21(7):1022-4.

210. Filazi A, Sinan I, Temamogullari F. Survey of the occurrence of aflatoxin M1 in cheeses produced by dairy ewe’s milk in Urfa city, Turkey. Ankara üniversitesi veteriner fakültesi dergisi. 2010;57(3):197-9.

211. Er B, Demirhan B, Onurdag FK, Yentur G. Determination of aflatoxin M1 level in milk and white cheese consumed in Ankara region, Turkey. Journal of Animal and Veterinary Advances. 2010;9(12):1780-4.

212. Hampikyan H, Bingol EB, Cetin O, Colak H. Determination of aflatoxin M1 levels in Turkish white, kashar and tulum cheeses. J Food Agric Environ. 2010;8(8):13-5.

213. Atasever MA, Adıgüzel G, Atasever M, Özturan K, Cops NA. Determination of Aflatoxin M1 levels in some cheese types consumed in Erzurum-Turkey. Kafkas Univ Vet Fak Derg. 2010;16:S87-S91.

214. Aksoy A, Yavuz O, Guvenc D, YK D, Terzi G, Celik S. Determination of Aflatoxin Levels in Raw Milk, Cheese and Dehulled Hazelnut Samples Consumed in Samsun Province, Turk. Kafkas Üniversitesi Veteriner Fakültesi Dergisi. 2010;16(1).

215. Atasever M, Adiguzel G, Atasever M, Özlü H, Özturan K. Occurrence of aflatoxin M1 in UHT milk in Erzurum-Turkey. Kafkas Univ Vet Fak Derg. 2010;16(Suppl A):S119-S22.

216. Mohamadi H, Alizadeh M. A study of the occurrence of aflatoxin M1 in dairy products marketed in Urmia, Iran. Journal of Agricultural Science and Technology. 2010;12(5):579-83.

217. Rohani FG, Aminaee MM, Kianfar M. Survey of aflatoxin M1 in cow's milk for human consumption in Kerman Province of Iran. Food Additives and Contaminants: Part B. 2011;4(3):191-4.

218. Panahi P, Kasaee S, Mokhtari A, Sharifi A, Jangjou A. Assessment of aflatoxin M1 contamination in raw milk by ELISA in Urmia, Iran. American-Eurasian Journal of Toxicological Sciences. 2011;3(4):231-3.

219. Movassagh MH. Presence of aflatoxin M1 in UHT milk in Tabriz (Northwest of Iran). Journal of Food Safety. 2011;31(2):238-41.

220. Maktabi S, Hajikolaie M, Ghorbanpour M, Pourmehdi M. Determination of aflatoxin M1 in UHT, pasteurized and GSM milks in Ahvaz (south-west of Iran) using Elisa. Global Veterinaria. 2011;7(1):31-4.

221. Buldu H, Koc AN, URAZ G. Aflatoxin M1 contamination in cow’s milk in Kayseri (central Turkey). Turkish Journal of Veterinary & Animal Sciences. 2011;35(2):87-91.

222. Ertas N, Gonulalan Z, Yildirim Y, Karadal F. A survey of concentration of aflatoxin M1 in dairy products marketed in Turkey. Food Control. 2011;22(12):1956-9.

223. El Khoury A, Atoui A, Yaghi J. Analysis of aflatoxin M1 in milk and yogurt and AFM1 reduction by lactic acid bacteria used in Lebanese industry. Food Control. 2011;22(10):1695-9.

224. Assem E, Mohamad A. A survey on the occurrence of aflatoxin M1 in raw and processed milk samples marketed in Lebanon. Food Control. 2011;22(12):1856-8.

225. Azizollahi Aliabadi M, Issazadeh K, Kazemi Darsanaki R, Laleh Rokhi M, Amini A. Determination of aflatoxin M1 levels in 1 white cheese samples by ELISA in Gilan province, Iran. Glob Vet. 2012;9(1):28-31.

226. Sepehr S, Amin J, Masoomeh G, Sahab S. Detection and occurance of aflatoxin M1 levels in milk and white cheese produce in Esfahan State Iran. Research Journal of Biological Sciences. 2012;7(5):225-9.

227. Mohamadi Sani A, Khezri M, Moradnia H. Determination of aflatoxin in milk by ELISA technique in Mashad (Northeast of Iran). International Scholarly Research Notices. 2012;2012.

228. Issazadeh K, Darsanaki R, Pahlaviani M. Occurrence of aflatoxin M1 levels in local yogurt samples in Gilan Province, Iran. Ann Biol Res. 2012;3(8):3853-5.

229. Rahimi E, Mohammadhosseini Anari M, Alimoradi M, Rezaei P, Arab M, Goudarzi M. Aflatoxin M1 in pasteurized milk and white cheese in Ahvaz, Iran. Global Veterinaria. 2012;9(4):384-7.

230. Nilchian Z, Rahimi E. Aflatoxin M1 in yoghurts, cheese and ice-cream in Shahrekord-Iran. World Appl Sci J. 2012;19(5):621-4.

231. Khoshnevis S, Gholampour Azizi I, Shateri S, Mousavizadeh M. Determination of the aflatoxin M1 in ice cream in Babol City (Northern, Iran). Glob Vet. 2012;8(2):205-8.

232. Tavakoli HR, Riazipour M, Kamkar A, Shaldehi HR, Nejad ASM. Occurrence of aflatoxin M1 in white cheese samples from Tehran, Iran. Food Control. 2012;23(1):293-5.

233. Behfar A, Khorasgani ZN, Alemzadeh Z, Goudarzi M, Ebrahimi R, Tarhani N. Determination of Aflatoxin M1 levels in produced pasteurized milk in Ahvaz City by using HPLC. Jundishapur Journal of Natural Pharmaceutical Products. 2012;7(2):80.

234. Rahimi E, Ameri M. A survey of aflatoxin M 1 contamination in bulk milk samples from dairy bovine, ovine, and caprine herds in Iran. Bulletin of Environmental Contamination and Toxicology. 2012;89:158-60.

235. Kocasari FS, Tasci F, Mor F. Survey of aflatoxin M1 in milk and dairy products consumed in Burdur, Turkey. International journal of dairy technology. 2012;65(3):365-71.

236. Kabak B, Ozbey F. Aflatoxin M1 in UHT milk consumed in Turkey and first assessment of its bioaccessibility using an in vitro digestion model. Food Control. 2012;28(2):338-44.

237. Zuheir I, Omar J. Presence of aflatoxin M1 in raw milk for human consumption in Palestine. Walailak J Sci Tech. 2012;9:201-5.

238. Behnamipour S, Arast Y, Mohammadian M. Occurence of aflatoxin M1 in two dairy products by ELISA in central part of Iran. Life Science Journal. 2012;9(3):1831-3.

239. Tabari M, Tabari K, Tabari O. Aflatoxin M1 determination in yoghurt produced in Guilan province of Iran using immunoaffinity column and high-performance liquid chromatography. Toxicology and Industrial Health. 2013;29(1):72-6.

240. Yosef T, Al-Julaifi M, Salah-El-Dein W, Al-Rizqi A. Assessment of aflatoxin M1 residues in raw cow milk at Al-Riyadh area with reference to some detoxification applications. Life Science Journal. 2013;10(1).

241. S AIAD A, HS AE. Aflatoxin M1 levels in milk and some dairy products in Alexandria city. Assiut Veterinary Medical Journal. 2013;59(139):93-8.

242. KAZEMI DR, AZIZOLLAHI AM, MOHAMMAD DCM. Aflatoxin M1 contamination in ice-cream. 2013.

243. Sani AM, Nikpooyan H. Determination of aflatoxin M1 in milk by high-performance liquid chromatography in Mashhad (north east of Iran). Toxicology and Industrial Health. 2013;29(4):334-8.

244. Tavakoli HR, Kamkar A, Riazipour M, Nejad ASM, Shaldehi HR. Assessment of Aflatoxin M-1 Levels by Enzyme-linked Immunosorbent Assay in Yoghurt Consumed in Tehran, Iran. Asian Journal of Chemistry. 2013;25(5):2836-8.

245. Ghaedi A, Mohamadi Sani A. Determination of AFM1 in milk by ELISA technique in quchan (North-east of Iran). International Scholarly Research Notices. 2013;7:337-40.

246. Mohajeri FA, Ghalebi SR, Rezaeian M, Gheisari HR, Azad HK, Zolfaghari A, et al. Aflatoxin M1 contamination in white and Lighvan cheese marketed in Rafsanjan, Iran. Food control. 2013;33(2):525-7.

247. Darsanaki RK, Mohammadi M, Kolavani MH, Issazadeh K, Aliabadi MA. Determination of aflatoxin M1 levels in raw milk samples in Gilan, Iran. Adv Stud Biol. 2013;5(4):151-6.

248. Tosun H, Ayyıldız T. Occurrence of aflatoxin M1 in organic dairy products. Quality assurance and safety of crops & foods. 2013;5(3):215-9.

249. Moosavy M, Roostaee N, Katiraee F, Habibi-Asl B, Mostafavi H, Dehghan P. Aflatoxin M1 occurrence in pasteurized milk from various dairy factories in Iran. International Food Research Journal. 2013;20(6):3351.

250. Kamkar A, Yazdankhah S, Mohammadi Nafchi A, Mozaffari Nejad AS. Aflatoxin M1 in raw cow and buffalo milk in Shush city of Iran. Food Additives & Contaminants: Part B. 2014;7(1):21-4.

251. Rahimi E. Survey of the occurrence of aflatoxin M1 in dairy products marketed in Iran. Toxicology and Industrial Health. 2014;30(8):750-4.

252. Rahimirad A, Maalekinejad H, Ostadi A, Yeganeh S, Fahimi S. Aflatoxin M1 concentration in various dairy products: evidence for biologically reduced amount of AFM1 in Yoghurt. Iranian journal of public health. 2014;43(8):1139.

253. Khodadadi M, Khosravi R, Allahresani A, Khaksar Y, Rafati L, Barikbin B. Occurrence of aflatoxin M1 in pasteurized and traditional cheese marketed in southern Khorasan, Iran. Journal of food quality and hazards control. 2014;1(3):77-80.

254. Kara R, Ince S. Aflatoxin M1 in buffalo and cow milk in Afyonkarahisar, Turkey. Food Additives & Contaminants: Part B. 2014;7(1):7-10.

255. ÖZTÜRK B, ÇELİK F, ÇELİK Y, KABARAN S, ZİVER T. To determine the occurence of aflatoxin M1 (AFM1) in samples of Cyprus traditional cheese (Halloumi): a cross-sectional study. Kafkas Üniversitesi Veteriner Fakültesi Dergisi. 2014;20(5).

256. Golge O. A survey on the occurrence of aflatoxin M1 in raw milk produced in Adana province of Turkey. Food Control. 2014;45:150-5.

257. Christofidou M, Kafouris D, Christodoulou M, Stefani D, Christoforou E, Nafti G, et al. Occurrence, surveillance, and control of mycotoxins in food in Cyprus for the years 2004–2013. Food and Agricultural Immunology. 2015;26(6):880-95.

258. Mwanza M, Abdel-Hadi A, Ali AM, Egbuta M. Evaluation of analytical assays efficiency to detect aflatoxin M1 in milk from selected areas in Egypt and South Africa. Journal of Dairy science. 2015;98(10):6660-7.

259. El-kest MM, El-Hariri M, Khafaga N, Refai MK. Studies on contamination of dairy products by aflatoxin M1 and its control by probiotics. J Glob Biosci. 2015;4(1):1294-312.

260. Elsayed MS, Abd El-Fatah EN. Prevalence of aflatoxin M1 in some milk products widely consumed by infants and children, marketed in Sharkia, Egypt. Global Vet. 2015;14(4):560-6.

261. Fallah AA, Barani A, Nasiri Z. Aflatoxin M1 in raw milk in Qazvin Province, Iran: a seasonal study. Food Additives & Contaminants: Part B. 2015;8(3):195-8.

262. Barikbin B, Allahresani A, Khosravi R, Khodadadi M. Detection of aflatoxin M1 in dairy products marketed in Iran. Health Scope. 2015;4(1).

263. Zanjani BR, Rahmani R, Sorkhabadi SMR, Aryan E, Oskouei Z, Sadeghi M, et al. A survey on aflatoxin M1 in raw milk of Fariman city, Khorasan province, Iran. Jundishapur Journal of Natural Pharmaceutical Products. 2015;10(2).

264. Rouhi R, Kazemi A, Jahromi AS, Zabetian H, Hakimelahi H, Yusefi A, et al. Levels of aflatoxin M1 in different types of milk collected in Jahrom, Iran, winter-spring 2013. Am J Anim Vet Sci. 2015;10(3):193-6.

265. Mason S, Arjmandtalab S, Hajimohammadi B, Khosravi Arsanjani A, Karami S, Sayadi M, et al. Aflatoxin M1 contamination in industrial and traditional yogurts produced in Iran. Journal of food quality and hazards control. 2015;2(1):11-4.

266. Mohajeri FA, Amiri A, Azad HK, Ahmadi Z, Asadollahi Z, Rezaeian M, et al. Occurrence of aflatoxin M1 in raw and pasteurized milk produced in Rafsanjan, Iran. Journal of Community Health Research. 2015;4(3):215-9.

267. Kocak P, GÖKSOY EÖ, Filiz K, Beyaz D, BÜYÜKYÖRÜK S. Occurrence of Aflatoxin M1 in flavored UHT milk. Ankara Üniversitesi Veteriner Fakültesi Dergisi. 2015;62(3):217-22.

268. Sarica DY, Has O, Tasdelen S, Ezer Ü. Occurrence of aflatoxin M1 in milk, white cheese and yoghurt from Ankara, Turkey markets. Biol Chem Res. 2015;2015:36-49.

269. Younis G, Ibrahim D, Awad A, El Bardisy M. Determination of aflatoxin M1 and ochratoxin A in milk and dairy products in supermarkets located in Mansoura City, Egypt. Adv Anim Vet Sci. 2016;4(2):114-21.

270. Hashemi M. A survey of aflatoxin M1 in cow milk in Southern Iran. Journal of food and drug analysis. 2016;24(4):888-93.

271. Dakhili M, Shalibeik S, Ahmadi I. Detection of aflatoxin M1 in milk from Qom (aried and semiaried) province of Iran. International Journal of Advanced Biotechnology and Research. 2016;7(3):1461-5.

272. Nikbakht MR, Lachiniyan S, Rahbar S, Oubari F, Rostami Z, Tajehmiri A. Aflatoxin M1 contamination in traditional yoghurts produced in Guilan province, Iran. Asian Journal of Pharmaceutical Research and Health Care. 2016:1-3.

273. Ghajarbeygi P, Palizban M, Mahmoudi R, Khaniki GJ, Pakbin B. Aflatoxin M1 contamination of cow’s raw milk in different seasons from Qazvin province, Iran. Journal of Biology and today's world. 2016;5(10):173.

274. Sohrabi N, Gharahkoli H. A seasonal study for determination of aflatoxin M1 level in dairy products in Iranshahr, Iran. Current medical mycology. 2016;2(3):27.

275. Mashak Z, Sohi HJ, Heshmati A, Nejad ASM. Assessment of AflatoxinM1 contamination in UHT flavored milk samples in Karaj, Iran. Iranian Journal of Pharmaceutical Research: IJPR. 2016;15(3):407.

276. Fallah AA, Fazlollahi R, Emami A. Seasonal study of aflatoxin M1 contamination in milk of four dairy species in Yazd, Iran. Food Control. 2016;68:77-82.

277. Taherabadi MS, Gharavi MJ, Javadi I, Alimohammadi M, Moghadamnia H, Mosleh N, et al. The level of Aflatoxin M1 in raw and pasteurized milk produced in Alborz Province, Iran. Jundishapur Journal of Natural Pharmaceutical Products. 2016;11(4).

278. Tajik H, Moradi M, Razavi Rohani S, Hadian M. Determination of aflatoxin M1 in pasteurized and UHT milk in West-Azerbaijan province of Iran. Journal of food quality and hazards control. 2016;3(1):37-40.

279. Özgören E, Kemal Seçkin A. Aflatoxin M1 contaminations in mouldy cheese. Mljekarstvo. 2016.

280. Sahin HZ, Celik M, Kotay S, Kabak B. Aflatoxins in dairy cow feed, raw milk and milk products from Turkey. Food Additives & Contaminants: Part B. 2016;9(2):152-8.

281. Omar SS. Aflatoxin M1 levels in raw milk, pasteurised milk and infant formula. Italian journal of food safety. 2016;5(3).

282. Tahoun AB, Ahmed MM, Abou Elez RM, AbdEllatif SS. Aflatoxin M1 in milk and some dairy products: level, effect of manufature and public health concerns. Zagazig Veterinary Journal. 2017;45(2):188-96.

283. Koutamehr ME, Akbari H, Akbari S, Hassanzadazar H. Aflatoxin M1 level in raw milk samples of Maragheh, Bonab and Malekan cities, East Azerbaijan province, Iran. Studia Universitatis" Vasile Goldis" Arad Seria Stiintele Vietii (Life Sciences Series). 2017;27(2):85-9.

284. Shahbazi Y, Nikousefat Z, Karami N. Occurrence, seasonal variation and risk assessment of exposure to aflatoxin M1 in Iranian traditional cheeses. Food Control. 2017;79:356-62.

285. Shokri H, Torabi S. The effect of milk composition, yeast‐mould numbers and seasons on aflatoxin M1 amounts in camel milk. Journal of Food Safety. 2017;37(2):e12300.

286. Sharifzadeh A, Ghasemi-Dehkordi P, Foroughi M, Mardanpour-Shahrekordi E, Ramazi S. Aflatoxin M1 contamination levels in cheeses sold in Isfahan Province, Iran. Osong public health and research perspectives. 2017;8(4):260.

287. Movassaghghazani M, Ghorbiani M. Incidence of aflatoxin M1 in human and cow milk in Kashan, Iran. Journal of food quality and hazards control. 2017;4(4):99-102.

288. Babolhavaegi HR, Afshar Y, Malekahmadi R, Hosseini MS, Norouzi HA, Zafar Mirmohamadi AR, et al. Determination of aflatoxin M1 in pasteurized and traditional milk in Hamadan Province, Iran. Journal of Environmental Health and Sustainable Development. 2018;3(2):504-8.

289. YILIDIRIM E, Macun HC, Yalcinkaya I, KOCASARI FŞ, Ekici H. Survey of aflatoxin residue in feed and milk samples in Kırıkkale province, Turkey. Ankara Üniversitesi Veteriner Fakültesi Dergisi. 2018;65(2):199-204.

290. ÖZTÜRK YILMAZ S, Altinci A. Incidence of aflatoxin M1 contamination in milk, white cheese, kashar and butter from Sakarya, Turkey. Food science and technology. 2018;39:190-4.

291. Madali B, Gulec A, Ayaz A. A survey of Aflatoxin M1 in different milk types in Turkey: risk assessment of children’s exposure. Progress in Nutrition. 2018;20(4):659-64.

292. Sakin F, Tekeli İO, Yipel M, Kürekci C. Occurrence and health risk assessment of aflatoxins and ochratoxin a in Sürk, a Turkish dairy food, as studied by HPLC. Food Control. 2018;90:317-23.

293. Hassan ZU, Al-Thani R, Atia FA, Almeer S, Balmas V, Migheli Q, et al. Evidence of low levels of aflatoxin M1 in milk and dairy products marketed in Qatar. Food control. 2018;92:25-9.

294. Shehab LM, El-Leboudy AA, Abo El-Makarem HS. Prevalence of Aflatoxins M1 and M2 in some curd dairy products. Alexandria Journal for Veterinary Sciences. 2019;61(1).

295. F. Abdallah M, Girgin G, Baydar T. Mycotoxin detection in maize, commercial feed, and raw dairy milk samples from Assiut City, Egypt. Veterinary sciences. 2019;6(2):57.

296. Khaneghahi Abyaneh H, Bahonar A, Noori N, Yazdanpanah H, Shojaee Aliabadi MH. Aflatoxin M1 in raw, pasteurized and UHT milk marketed in Iran. Food additives & contaminants: part B. 2019;12(4):236-44.

297. Abyaneh HK, Bahonar A, Noori N, Yazdanpanah H, AliAbadi MHS. Exposure to aflatoxin M1 through milk consumption in Tehran population, Iran. Iranian Journal of Pharmaceutical Research: IJPR. 2019;18(3):1332.

298. Mahmoodi Maymand M, Mazaheri M, Talebi Mehrdar M. Determination of Aflatoxin M1 in pasteurized liquid and powdered milk products imported to Iran. Iranian Journal of Toxicology. 2019;13(2):19-23.

299. Nejad ASM, Heshmati A, Ghiasvand T. The occurrence and risk assessment of exposure to aflatoxin M1 in ultra-high temperature and pasteurized milk in Hamadan province of Iran. Osong public health and research perspectives. 2019;10(4):228.

300. Ansari F, Pourjafar H, Christensen L. A study on the aflatoxin M1 rate and seasonal variation in pasteurized cow milk from northwestern Iran. Environmental monitoring and assessment. 2019;191:1-6.

301. Acaroz U. Assessment of aflatoxin M1 levels in tulum and white cheeses in afyonkarahisar, turkey. Fresenius Environ Bullet. 2019;28(11a):8663-8.

302. Eker FY, Muratoglu K, Eser AG. Detection of aflatoxin M 1 in milk and milk products in Turkey. Environmental monitoring and assessment. 2019;191:1-8.

303. Çetin B, Özcan Y, Aloğlu HŞ, Becker A. Determination of Seasonal Distribution of. Arch Lebensmittelhyg. 2019;70:17-22.

304. El-Tawab A, El-Hofy F, El-Diasty E, Abo-Hamdah E, El-Hayat M. Prevalence of aflatoxin m1 and molecular studies on some food born fungi isolated from milk and dairy products. Adv Anim Vet Sci. 2020;8(3):305-11.

305. Ahmed AE, AL-Kahtani MM, El-Diasty EM, Ahmed AS, Saber H, Abbas AM, et al. Diversity of Toxigenic Molds and Mycotoxins Isolated from Dairy Products: Antifungal Activity of Egyptian Marine Algae on Aspergillus and Candida Species. Journal of Pure & Applied Microbiology. 2020;14(1).

306. Ismaiel AA, Tharwat NA, Sayed MA, Gameh SA. Two-year survey on the seasonal incidence of aflatoxin M1 in traditional dairy products in Egypt. Journal of food science and technology. 2020;57:2182-9.

307. Ahmadi E. Potential public health risk due to consumption of contaminated bovine milk with aflatoxin M1 and Coxiella burnetii in the West of Iran. International Journal of Dairy Technology. 2020;73(3):479-85.

308. Nejad ASM, Heshmati A, Ghiasvand T. The occurrence and risk assessment of aflatoxin M1 in Cheeses samples from Hamadan, Iran. Iranian Journal of Pharmaceutical Research: IJPR. 2020;19(4):44.

309. Heshmati A, Mozaffari Nejad ASM, Ghyasvand T. The occurrence and risk assessment of aflatoxin M in yoghurt samples from Hamadan, Iran. The open public health journal. 2020;13(1).

310. Daou R, Afif C, Joubrane K, Khabbaz LR, Maroun R, Ismail A, et al. Occurrence of aflatoxin M1 in raw, pasteurized, UHT cows’ milk, and dairy products in Lebanon. Food control. 2020;111:107055.

311. El Tawila M, Sadeq S, Awad AA, Serdar J, Madkour MHF, Deabes MM. Aflatoxins contamination of human food commodities collected from Jeddah markets, Saudi Arabia. Open Access Macedonian Journal of Medical Sciences (OAMJMS). 2020;8(E):117-26.

312. Murshed S. Evaluation and assessment of aflatoxin M1 in milk and milk products in Yemen using high-performance liquid chromatography. Journal of Food Quality. 2020;2020:1-8.

313. Adam A, Aly S, Sayed R, Saad M. Occurrence of Aflatoxin M1 in Cheese and Yoghurt Marketed at El-Fayoum Province, Egypt. International Journal of Dairy Science. 2021;16:146-52.

314. Jafari K, Fathabad AE, Fakhri Y, Shamsaei M, Miri M, Farahmandfar R, et al. Aflatoxin M1 in traditional and industrial pasteurized milk samples from Tiran County, Isfahan Province: A probabilistic health risk assessment. Italian Journal of Food Science. 2021;33(SP1):103-16.

315. Onmaz NE, Gungor C, Al S, Dishan A, Hizlisoy H, Yildirim Y, et al. Mycotoxigenic and phylogenetic perspective to the yeasts and filamentous moulds in mould-matured Turkish cheese. International Journal of Food Microbiology. 2021;357:109385.

316. Esam RM, Hafez RS, Khafaga NIM, Fahim KM, Ahmed LI. Assessment of aflatoxin M1 and B1 in some dairy products with referring to the analytical performances of enzyme-linked immunosorbent assay in comparison to high-performance liquid chromatography. Veterinary World. 2022;15(1):91.

317. TAŞÇI F, EROL Z, KOCASARI FŞ. Investigation of Aflatoxin M1 Residue in Raw Cow Milk Samples in Burdur.

318. Khalifa MI, Sallam KI, Kasem NG. Detection of Aflatoxin M1 in the Milk of Naturally Grazed and on-farm-fed Camels. Journal of Advanced Veterinary Research. 2023;13(3):521-5.

319. Youssef NH, El Gammal MH, Altaie HA, Qadhi A, Tufarelli V, Losacco C, et al. Mycotoxins in milk: Occurrence and evaluation of certain detoxification attempts. Food Science & Nutrition. 2023.

320. Hamad GM, El-Makarem HSA, Allam MG, El Okle OS, El-Toukhy MI, Mehany T, et al. Evaluation of the Adsorption Efficacy of Bentonite on Aflatoxin M1 Levels in Contaminated Milk. Toxins. 2023;15(2):107.
